# Have We Been Measuring Migrant Wellbeing all Wrong? Conceptualizing Migrant Wellbeing: A Systematic Review

**DOI:** 10.1007/s10903-025-01773-z

**Published:** 2025-09-10

**Authors:** Salsawi Feleke Debela, Sheenagh McShane, Lauren Carpenter, Celia McMichael, Ankur Singh, Karen Block

**Affiliations:** 1 Melbourne School of Population and Global Health, University of Melbourne, Melbourne, Australia; 2 University of Sydney School of Dentistry, University of Sydney, Sydney, Australia; 3 Charles Perkins Centre, University of Sydney, Sydney, Australia; 4 School of Geography, Earth and Atmospheric Sciences, The University of Melbourne, Melbourne, Australia; 5 Centre for Epidemiology and Biostatistics, Melbourne School of Population and Global Health, University of Melbourne, Melbourne, Australia

**Keywords:** Wellbeing, Migrant, Migration, Wellbeing tools, Migrant wellbeing, Wellbeing measurement

## Abstract

Wellbeing is a widely used concept yet lacks a universal definition and standardized measurement. Migrants, especially those forcibly displaced, face challenges that impact their quality of life and wellbeing. To understand how the wellbeing of people who migrate from low/middle-income countries to middle/high-income countries has been conceptualized, defined, and measured. Four databases (MEDLINE, PubMed, EMBASE, and PsycINFO) were searched. Two researchers independently screened all articles, with narrative synthesis and the Consensus-based Standards for the selection of health Measurement Instruments (COSMIN) checklist used for analysis. The search returned 5,610 articles, with 126 included in this review. Of these 126 articles, 89 did not explicitly define nor conceptualise wellbeing. Forty-three measurement tools were used to measure the wellbeing of 281,478 migrants in more than 35 countries. Seven tools were used in three or more articles, two of which were not specifically designed for wellbeing measurement. Two tools Personal Wellbeing Index (PWI) and WHO Quality of life Brief (WHOQoL-BREF) had satisfactory COSMIN scores. Fewer than half (47.6%) of the articles reported translating tools into respondents’ languages. Tools designed and developed in the context of Global North, middle-class populations, might not accurately measure wellbeing in migrant groups. This review highlights critical gaps in the way migrant wellbeing is measured. Without culturally informed tools, understanding of migrant wellbeing will remain fragmented, limiting development of effective and equitable public health interventions and policies.

**Supplementary Information:**

The online version contains supplementary material available at 10.1007/s10903-025-01773-z.

## Introduction

We live in the “age of migration” [1] where millions move across national and international borders each year both as voluntary migrants or as forcibly displaced populations searching for safety and security. In 2024, 3.6% of the world’s population (281 million) lived outside their country of origin [2]. Many migrants, especially those who are forcibly displaced by war, violence and persecution, face challenges pre-, during, and post-migration. Post-migration stressors include transitioning into a new culture and new systems, shifts in cultural identity, acculturative stress, and poor mental health [3]. Migrants can also experience loss of culture, social capital, customs, religious activities and social support; and legal concerns and cultural bereavement [4]. Most of these challenges can adversely affect their lives and wellbeing [5].

Migrants differ from both ‘host’ and ‘home’ populations. Sayad [6] argues, for example, that migrants are neither part of the ‘home’ nor ‘host’ society; they are ‘double absent’, uprooted and disconnected from their origin and not full members in the host country. Contrastingly, Veikou and Siapera, argue that migrants have ‘multiple presence’; in the age of fast internet and social media, migrants simultaneously ‘belong’ to both their ‘home’ and ‘host’ society [7]. Migrants can often find themselves juggling between ‘home’ and ‘host’ country, negotiating whether they belong to, ‘home’, ‘host’ or ‘nowhere’ resulting in a feeling of being stuck in liminal space [25, 26]. This can affect their wellbeing and as a result, their wellbeing may differ from both home and host populations.

Wellbeing is a widely used concept but there is no universally agreed definition or measurement tool [10]. Broadly, “wellbeing refers to all of the various types of evaluations, both positive and negative, that people make of their lives” [11, p. 399]. It is generally conceived as the quality of a person’s life, including living conditions, subjective life satisfaction, materials and health [12]. Positive wellbeing may be considered an indicator of successful settlement, healthy functioning and integration into society [13].

There are over 1,200 wellbeing measurement tools [14], with a significant increase in the scientific study of wellbeing over the past decades [15]. However, this proliferation has led to diverse conceptions and wellbeing measurement tools [16]. As wellbeing researcher Cummins has noted, “[t]he unfortunate result [of this proliferation] is a confused and massive literature” that has resulted in an intellectual maze [17, p. 518].

Much wellbeing research, particularly that concerning forced migrants, is focused on mental illness/disorder [14, 15]. Aspects of migrant and refugee subjective wellbeing have been investigated somewhat less frequently, despite being purportedly useful indicators of quality of life and successful integration [20]. Researchers who have assessed migrant wellbeing have used a range of tools. However, despite recognition that psychological and wellbeing evaluations are influenced by culture [21], many tools have not been developed appropriately for use in migrant populations [22]. Almost all wellbeing measurement tools have been developed in English, based on Western, individualistic cultural norms [23]. English is not the first language of many migrants and refugees, and many come from cultural backgrounds with collectivistic sociocultural orientations [24]. Tools may be problematic to use in languages other than English and/or lack cross-cultural validity when used to assess and measure wellbeing in different cultural groups [25].

While some wellbeing measurement tools have been validated in migrant population groups, differences in language, culture, and socialization about expressing, responding and sharing thoughts and feelings have hampered cross-cultural validations [13, 21]. One issue is lack of simple equivalence between words, phrases and expressions in different languages and cultures [25]. As Edward Sapir, considered the founder of ethnolinguistics, argues, “[n]o two languages are ever sufficiently similar to be considered as representing the same social reality” [26, p. 209].

Most (systematic) reviews of wellbeing measurement tools have focused on evaluating measurement and psychometric properties of tools in general [12, 27]. Some have considered specific populations such as farmworkers [28]; older adults in aged care [29]; people with intellectual disability [30]; and children [31]. To date, no systematic review has evaluated the properties, suitability, and appropriateness of wellbeing measurement tools for migrant populations.

The aim of this review is to assess and understand how the wellbeing of people who migrate from low- and/or middle-income countries (LMIC) to middle- and/or high-income countries (MHIC) has been conceptualized, defined, and measured. LMIC and MHIC are the most common origins and destinations of migrants, respectively [2]. Specifically, this review address three research questions. First, how has wellbeing been defined and conceptualized in studies that measure migrant wellbeing? Second, what tools have been used to measure the wellbeing of migrants? Third, how appropriate are these tools for measuring migrant wellbeing?

## Methods

The protocol for this systematic review is registered on PROSPERO (https://www.crd.york.ac.uk/prospero/display_record.php? ID=CRD42023445945).

### Search Strategy

The search strategy (Table 1) was run in four databases (MEDLINE, PubMed, EMBASE, PsycINFO).

**Table 1:** Search strategy

	Search terms
1	Migrant*.mp. or “international migration”.mp. or Refugee*.mp. or refugees.mp. or political refugee.mp. or transient*.mp. or “transient and migrants”.mp. “emigrants and immigrants”.mp. or “emigration and immigration”.mp. or immigrant*.mp. Or asylum seeker*.mp. or political asylum seeker*.mp. displaced person*.mp. or “culturally and linguistically diverse person”.mp. or CALD.mp. or resettlement.mp.
2	Well-being.mp. or wellbeing.mp. or well being.mp. or psychological wellbeing.mp
3	(scale* or measure* or assessment* or test or tests or survey* or screen*).mp. or psychological tests/or patient health questionnaire/.mp. or “surveys and questionnaires”/or health surveys/or patient health questionnaire/
4	Publication date parameters January 2000 to July 2023

### Inclusion-exclusion Criteria

Articles needed to be original research using quantitative or mixed methods and published in English. For feasibility, and to ensure the review focused on relatively recent research, only research published since 2000 was included. Included articles focused on the wellbeing of international migrants (including first- and second-generation migrants, refugees and asylum seekers) who had migrated from LMIC to MHIC.

Articles were excluded if they focused on internally displaced persons (IDPs), rural-urban (within country) migrants and return migrants, as issues concerning translation across cultures and languages are likely to be less pertinent for these migrant groups. Articles focused on international students, tourists, children, resettlement in LMIC or people living in refugee camps (because they are in a temporary setting) were also excluded. Qualitative or conceptual studies, systematic/scoping/rapid/narrative reviews, pre-prints, unpublished or non-peer reviewed articles, protocols, grey literature and those focused specifically on mental illness (e.g. depression, anxiety, PTSD) were also excluded.

### Screening Process

Screening was undertaken independently by two researchers (SFD, SM) according to the inclusion-exclusion criteria using the online platform Covidence. After importing the articles to Covidence, the screening process involved three stages: title screening, abstract screening, and full text review. Disagreements were resolved by discussion between reviewers and other members of the authorship team.

### Data Extraction

A three-section data extraction tool was developed: 1/publication details; 2/study details (study design, recruitment method, number of participants, gender, age and national/ethnic identity of participants, migrant generation and type of migrant); 3/wellbeing measures (wellbeing definition, single/multi-item tool, number of tools used, name of tool/s, translation of tools, and study limitations). Data were extracted by one researcher (SFD) with a random 10% of articles checked by a second reviewer (SM).

### Evidence Synthesis

This review contains two main syntheses: (1) a narrative synthesis of 126 articles; and (2) Consensus Based Standards for the Selection of Health Measurement Instruments (COSMIN) analysis of six multi-item wellbeing measurement tools that featured in three or more articles.

### Narrative Synthesis

Narrative synthesis is an appropriate method for presenting a comprehensive overview of existing research in a field through systematic review [32]. Thus, to understand conception, definition, and measurement of migrant wellbeing in the last 23.5 years, a narrative synthesis of 126 articles included in this review, supported by summary tables and charts, is presented.

### COSMIN Analysis

The COSMIN checklist was first developed to objectively assess measurement properties of patient reported outcome measures (PROMs), but can also be used effectively to assess the measurement properties of quantitative tools [33]. We used the checklist to critically appraise the properties of wellbeing measurement tools. We applied the COSMIN checklist for the six most used multi-item tools. To help us evaluate the tools, we used the original tool development articles/validation studies and manuals. Tool authors were contacted where more information was needed. These six tools have been used by 65 studies included in this review. To supplement the COSMIN analysis, we also searched PubMed and Google Scholar for subsequent cross-cultural validation studies for the selected tools.

The COSMIN checklist has nine measurement properties, content validity, structural validity, internal consistency, cross-cultural validity/measurement invariance, reliability, measurement error, criterion validity, hypotheses testing for construct validity, and responsiveness, which are used to assess the quality of outcome measurement instruments. The guideline ranks these properties based on their level of relevance.

Content validity is ranked as most important, as it is crucial to ensure a measurement tool is relevant, complete and understandable before being administered. The internal structure of the tool, which includes structural validity, internal consistency and cross-cultural validity, is ranked as second most important. The other five characteristics (reliability, measurement error, criterion validity, hypotheses testing for construct validity and responsiveness) are ranked as less critical [34].

COSMIN uses the ‘worst score counts’ principle, i.e. the score assigned to a measurement instrument is the lowest of all the property scores, not the average. This principle is based on the understanding that “poor methodological aspects of a study cannot be compensated by other strong aspects” [33, p. 15]. The rating was done independently by two researchers (SFD, SM) and the final ratings was based on consensus reached among the reviewers.

## Results

**Fig. 1:**
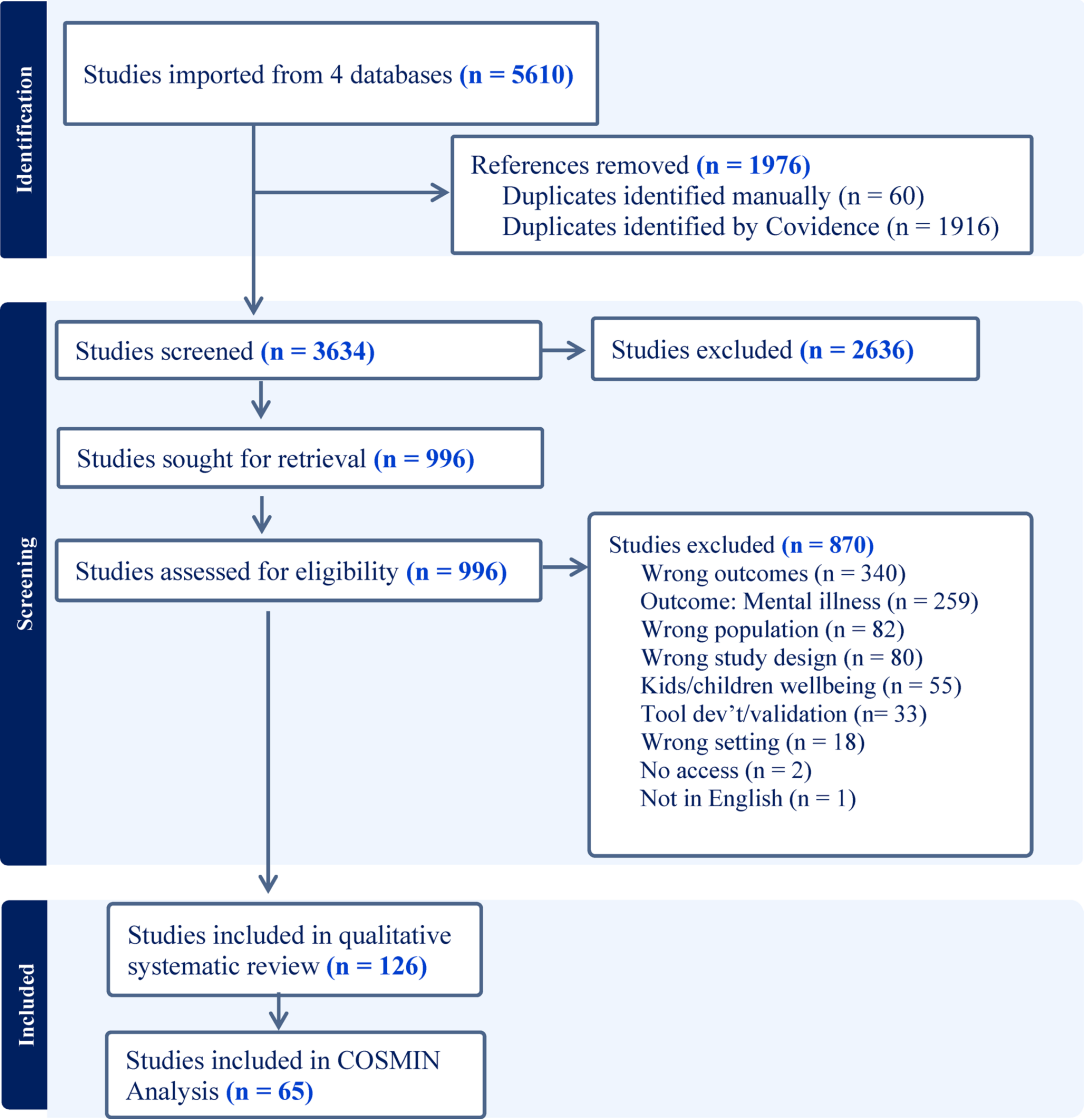
PRISMA diagram

The literature search located 5,610 articles, which were imported to Covidence^1^; 1,976 duplicates were removed, leaving 3,634 articles for screening. Following title and abstract screening of 3,634 articles, disagreements were discussed and resolved by the authorship team. This left 996 articles for full text review, of which 126 progressed to data extraction (see Fig. 1). The 126 articles cover 281,478 migrants, aged 15 to 79 + years, living in more than 35 middle- and high-income countries (noting that some reported regions rather than countries).

### Study Characteristics

Two-thirds of the articles, 85/126(67.5%), included in this review were conducted in ten countries: USA 23(18.3%), Israel 13(10.3%), Germany 8(6.3%), Spain 8(6.3%), Canada 7(5.6%), Sweden 6(4.8%), UK 5(4%), Australia 5(4%), Chile 5(4%), and the Netherlands 5(4%). The remaining 41(32.5%) studies were conducted in more than 25 other countries. Most migrants originated from four regions: Africa (e.g. sub-Sahara, North Africa), Asia (e.g. India, China), Middle East (e.g. Afghanistan, Iraq) and Eastern Europe (e.g. Former Soviet Union, Romania). For the full list of countries and other details of the study characteristics refer to Supplementary File, Table 1.

Just over half of the articles, 69/126(54.8%), described their study population as immigrants (without specifying whether their migration was voluntary or forced). Others, 41(32.5%), specified that their respondents were involuntary/forced migrants (including refugees, asylum-seekers, illegal and undocumented migrants). The remining 16(12.7%) were articles focusing on voluntary migrants (including labour migrants and marriage migrants).

Forty-three tools were used to measure migrant wellbeing (see Table 2 for the list of tools and see Supplementary File Table 1, for the full list of studies that used these tools). The two most used tools were Diener’s Satisfaction with Life Scale (SWLS) and General Life Satisfaction (GLS), a single item measure, each was reported in 23 different articles either alone or in conjunction with other wellbeing measurement tools. Most articles, 111/126(88.1%) reported using only one tool to measure wellbeing, 13(10.3%) reported two tools, and 2(1.6%) reported three. Most used multi-item tools, 103/126(81.7%), whilst 23(18.3%) used single item tools (generally large-scale multi-domain surveys).

**Table 2:** List of tools reported in the 126 articles

	Tools name	Abbreviations	Frequency
1	General Life Satisfaction (AKA single item)	GLS	23
2	Satisfaction With Life Scale	SWLS	23
3	WHO Quality of life Brief	WHOQoL-BREF	19
4	Well-being Index (WHO-5)	WHO-5	13
5	Ryff’s Psychological Well-Being Scale	Ryff	6
6	General Health Questionnaire (GHQ-12)	GHQ-12	4
7	Personal Wellbeing Index	PWI	4
8	Interpersonal, Community, Occupational, Physical, Psychological, and Economic Wellbeing Scale	I COPPE	2
9	Scale of Positive and Negative Experience	SPANE	2
10	Short form 12 item version 2	SF-12v2	2
11	Social Production Function Instrument for the Level of Well-being short	SPF-ILs	2
12	Social Wellbeing Scale	SWS	2
13	Warwick-Edinburgh Mental Wellbeing Scale (WEMWBS)	WEMWBS	2
14	3-item Life Satisfaction Scale developed by Bachman, Kahn, Davidson and Johnston (1967)	LSS-3	1
15	Questionnaire developed by Bradburn (1969)	Bradburn	1
16	Centre for Epidemiological Studies Depression Scale (CES-D)	CES-D	1
17	Control, Autonomy, Self-Realization and Pleasure	CASP-19	1
18	Differential Emotions Scale	DES	1
20	EUROHIS-QOL 8-item Index	EUROHIS-QOL	1
21	EuroQol EQ-5D	EQ-5D	1
22	Flourishing Scale (FS)	FS	1
23	Index of Life Satisfaction	ILS	1
24	Index of Well-Being by Campbell	IW-Campbell	1
25	Life Satisfaction Index-Z developed by Neugarten et al. (1961)	LSIZ	1
26	Manchester Short Assessment of Quality of Life	MANSA	1
27	Mental Health Continuum Short Form (MHC-SF)	MHC-SF	1
28	Mental Health Inventory (MHI	MHI	1
29	NEI & PEI (Negative & Positive Experience Index)	NEI & PEI	1
30	Positive and negative affect scale developed by UK office of national statistics	PNAS-UK	1
31	Satisfaction with Life Areas (SLA) scale	SLA	1
32	Short Form Survey Instrument (SF-36)	SF-36	1
33	Spiritual Well-Being Scale (SWBS)	SWBS	1
34	Subjective Happiness Scale	SHS	1
35	Visual Analogue Scale (VAS)	VAS	1
36	WHO (10) Well-Being Index	WHO-10	1
37^a^	Created by researchers (5 articles)		5
38^b^	Adapted existing tool (2 articles)		2

^a, b,^ The last two are categories (not tool names) that combine 7 articles. Because some studies used multiple tools, the total frequency of tools used, 134, is greater than the number of studies, 126.

Fewer than half of the articles, 60/126(47.6%) reported translating wellbeing measurement tools into respondents’ languages. Translation methods included using a validated version of the translated tool; using translation and back translation; using bilingual data collectors to translate the tool during data collection; and in some cases, researchers themselves undertaking translations. No information was provided about translation in 53(42.1%) articles, and 13(10.3%) reported using the English version of the tool without translation.

From the 60(47.6%) studies that reported translation, the most translated tools were WHOQoL-BREF (12/60, 20%), SWLS (12/60, 20%), WHO-5 (8/60, 13.3%) and GHQ-12, Ryff and GLS, each used three times (5%).

Of the 60(47.6%) that reported translating the tools, only 25(19.8%) studies reported using validated version of the translated tools. The most used validated tools were WHOQoL-BREF (9/25, 36%), WHO-5 (5/25, 20%), and SWLS (5/25, 20%). The full list of studies that used translated and validated translations can be found in Supplementary File Table 3.

### Defining and Conceptualising Wellbeing

Of the 126 included articles, 37(29.4%) defined wellbeing while 89(70.6%) did not give an explicit operational definition of the construct. Among those that defined wellbeing, five distinct concepts were identified: subjective wellbeing (SWB), quality of life (QOL), psychological wellbeing (PWB), life satisfaction (LSF), and social wellbeing (SOWB) (see Supplementary File Table 2, which details the definitions given in articles, compared with the definitions on which the tools used are based). For this review, we use ‘wellbeing’ as an umbrella term to include all these definitions.

- Subjective wellbeing “refers to all of the various types of evaluations, both positive and negative, that people make of their lives” [11, p. 399]. Twenty-one articles adopted the SWB definition, using 12 different tools to measure it. Seventeen articles used tools aligned with that definition and five reported using tools that did not align with their definition of SWB (one reported use of more than one tool).
- Quality of life “refers to the degree to which a person’s life is desirable versus undesirable, often with an emphasis on external components, such as environmental factors and income” [11, p. 401]. Seven articles used the QOL definition, of which four used the WHOQoL-BREF to measure it. In all seven, the definition and the tools used were aligned.
- Psychological wellbeing refers to “perception of engagement with existential challenges of life” [15, p. 1007]. Six articles used the PWB definition, of which three used Ryff’s Psychological Wellbeing Scale. In three cases the tools used and the definitions did not align, while five of the tools used and the definition given aligned (two articles reported use of more than one tool).
- Life satisfaction “represents a report of how a respondent evaluates or appraises his or her life taken as a whole” [11, p. 401]. Two articles used this definition. In both, the tools and definitions were aligned.
- Social wellbeing is defined as the function of five social dimensions: coherence, integration, actualization, contribution and acceptance [35, p. 133]. One article used this definition and tool aligned with the definition.

### COSMIN Results

**Fig. 2:**
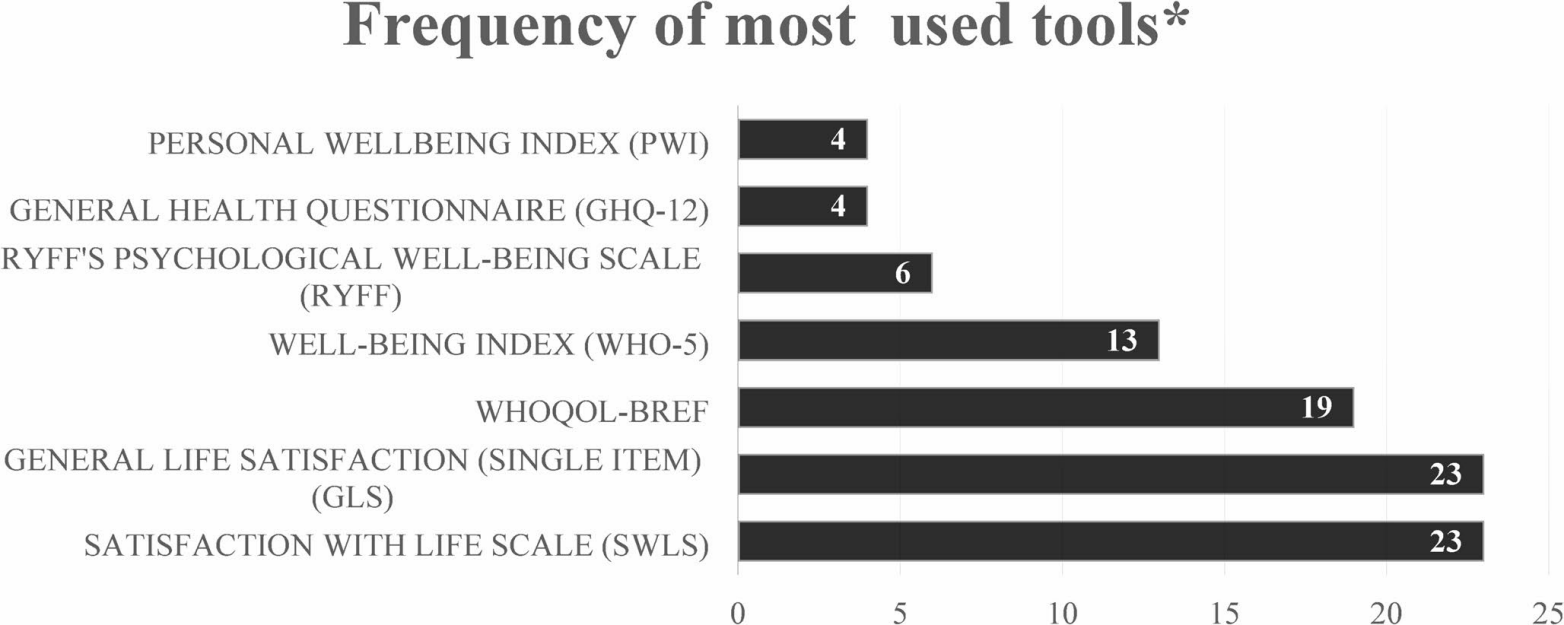
Frequency with which most commonly used tools were used. (*some articles used more than one tool, thus the total frequency of tools is greater than the number of articles included.)

We selected the most commonly used tools for COSMIN analysis. Seven tools were used three or more times across 88 different articles: PWI, GHQ-12, Ryff, WHO-5, WHOQoL-BREF, GLS and SWLS (Fig. 2). We did not include the GLS scale, in the COSMIN analysis, as single item tools cannot be analysed using the checklist. Thus, the COSMIN checklist was applied to six tools used in 65(51.6%) articles. The properties of the most used wellbeing tools are summarized and presented in Table 3.

The most commonly used tools for measuring migrant wellbeing were the GLS and SWLS, developed in 1976 and 1985 respectively. We also looked separately into the tools used to measure the wellbeing of voluntary and involuntary/forced migrants. When the mode of migration was specified, WHO-5 (11/45), WHOQoL-BREF (10/45) and PWI 3/45) were the most used for measuring wellbeing of involuntary/forced migrants; and SWLS (5/18), GLS (5/18) and WHO-5 (2/18) were the most common for voluntary migrants.

Of the seven commonly used tools, the newest, PWI developed in 2002, was the only tool to have been consistently updated. All had very good internal consistency, with Cronbach alpha ≥ 0.8. All were developed in the Global North, however WHOQoL-BREF was tested in 18 locations, including countries in the Global South. Two tools (GHQ-12 and WHO-5) that were designed as screening tools for psychiatric disorders and depression, respectively, were not designed to measure wellbeing yet were used for that purpose (see Table 3).

From the 65 studies included in the COSMIN review, 43/65(66.2%) reported sample specific internal consistency while 10/65(15.4%) reported non sample specific and 12/65(18.5%) did not report internal consistency for the tools they used. Similarly, 18/65(27.7%) reported sample specific validity and 29/65(44.6%) reported non sample specific, while 18/65(27.7%) did not repot validity for the instruments they used. Internal consistency and validity vary across sample groups and cultures. Reporting it, therefore indicates accuracy and cultural appropriateness of the tools used [36].

**Table 3:** Summary and properties of the most commonly used tools to measure migrant wellbeing in articles included in current review

Tool	Year	Country of development	Rationale	Sample group	Wellbeing definition	Conceptualisation	Domains	Total questions	Cronbach alpha
**General Life Satisfaction (GLS)**	1976 [37]	USA	To measure quality of life and wellbeing[38]	5422 North American adults[37]	How satisfied are you with your life as a whole these days? (7-point scale: Completely satisfied … completely dissatisfied)[37]	Subjective wellbeing	Single-item scale	1	
**Satisfaction with life scale (SWLS)**	1985[39]	USA	To measure life satisfaction as a cognitive-judgemental process. [39]	339 college students and 53 elderly people[39]	Life satisfaction as a global assessment of a person’s quality of life according to chosen criteria. [39]	Subjective wellbeing	Global life satisfaction[16]	5	0.87[16]
**General Health Questionnaire (GHQ-12)**	1988[40]	England	Screening test to detect psychiatric disorders. [40]	Psychiatric patients in London. [40]	‘Wellbeing’ is not mentioned/defined in the GHQ user guide. [40]	na	Positive and negative affect[13]	12	0.85[13]
**Ryff’s Psychological Wellbeing Scale (Ryff)**	1989[41]	USA	To develop a theoretically grounded measure of wellbeing. [41]	321 relatively healthy, well-educated, financially comfortable respondents[41]	Wellbeing is a function of six domains: self-acceptance, positive relations with others, autonomy, environmental mastery, purpose in life, and personal growth. [41]	Psychological wellbeing	Six dimensions: Autonomy, environmental mastery, personal growth, positive relations with others, purpose in life, self-acceptance. [41]	Parent scale = 120; (84, 54, 42, & 18 item versions)[42]	0.86–0.93[16]
**WHO-5**	1998[43]	Europe	To provide a screening tool for patients showing signs of depression. [43]	437 psychiatric patients in 4 European cities[43]	Wellbeing is equated with mental health[44] and measured as a function of five domains: Cheerfulness, calmness, activity, rest and interest. [43]	Mental wellbeing[45]	Cheerfulness, calmness, activity, rest and interest[43]	5	0.69–0.92[13]
**WHOQoL-BREF**	1998[46]	Europe	To develop an international measure of quality of life. [46]	4,802 respondents for pilot and 4101 for testing in 18 culturally diverse field centres around the world[46]	“individuals’ perceptions of their position in life in the context of the culture and value systems in which they live and in relation to their goals, expectations, standards and concerns”. [46]	Quality of life	**Six domains** (physical capacity, psychological, level of independence, social relationships, environment, and spiritual/religion/personal belief) and 24 facets[46]	26	0.68–0.88[16]
**Personal Wellbeing Index (PWI)**	2002[25]	Australia	To measure subjective quality of life and wellbeing.[14]	2000 people that collectively represented the national population of Australia[25]	Subjective wellbeing (SWB) refers to a person’s overall sense of wellbeing, happiness, and life satisfaction. [14]	Subjective wellbeing	**Seven domains**: standard of living, health, achieving in life, relationships, safety, community connectedness, and future security. [14]	7	0.80–0.9[13]

### Characteristics of the Wellbeing Measurement Tools

We evaluated the six most commonly used multi-item tools (PWI, GHQ-12, Ryff, WHO-5, WHOQoL-BREF and SWLS) using COSMIN. As noted earlier, the COSMIN guidelines hierarchically rank tool properties. Four of the six tools scored ‘doubtful’ on the most important COSMIN property, content validity. Content validity can be assessed in two population groups: patients and experts. Developers of the PWI consulted patients, whilst developers of the WHOQoL-BREF consulted patients and experts. For SWLS, Ryff, GHQ-12 and WHO-5, it is not clearly reported whether patients or experts were consulted during tool development.

The second most important property, internal structure of the instruments, includes structural validity, internal consistency and cross-cultural validity. All tools scored ‘very good’ for internal consistency. However, four tools (apart from WHOQoL-BREF and PWI) scored ‘inadequate’ or ‘doubtful’ for the other two properties. Cross-cultural validity is an important characteristic of a tool for the population group of interest (migrants). It is vital to evaluate whether tools created in one culture are applicable, meaningful and equivalent in another culture [47]. However, SWLS and Ryff scored ‘inadequate’ while GHQ-12 and WHO-5 scored ‘doubtful’. These tools were created based on testing and validations done in only homogeneous cultural group. On the other hand, PWI and WHOQoL-BREF scored ‘adequate’ and ‘very good’, respectively, because they were tested and validated in different cultural groups.

For the tools that scored, ‘inadequate’ or ‘doubtful’ in cross cultural validation (SWLS, Ryff, GHQ-12 and WHO-5), we tried to search for subsequent validation studies in different language and cultural groups. Through our search in PubMed and Google Scholar we found 40 cross-cultural and language validation studies for these four tools (See Supplementary File Table 5). Thus, despite the low COSMIN score, these tools have since been tested and validated in different cultural and language groups.

The third ranked set of characteristics are reliability, measurement error, criterion validity, construct validity and responsiveness. For reliability (the degree of being free of measurement error), the WHOQoL-BREF and PWI were rated as ‘very good’, because they used heterogeneous samples for testing and validation studies. The other four tools were rated as ‘inadequate’ or doubtful’, because their test and validity samples were homogenous. When examining measurement error, the PWI was rated ‘very good’, SWLS was rated ‘doubtful’ and the other four tools were rated ‘adequate’. All tools were rated ‘very good’ for criterion validity and ‘adequate’ or ‘very good’ for construct validity. Responsiveness (the ability of a tool to detect change over time) is assessed using four approaches: comparison with gold standard, comparison with other instruments, comparison between subgroups, and comparison before and after intervention. In the current analysis, tools were not assessed for ‘before and after intervention’ because none of the articles involved interventions; they only measured wellbeing at one point in time. All six tools performed well across the three responsiveness categories, except in one case where SWLS scored ‘inadequate’.

Thus, based on the COSMIN analysis, four of the most frequently used tools (SWLS, Ryff, WHO-5 and GHQ-12) performed poorly on two out of the three most important characteristics: content validity and internal structures. PWI and WHOQoL-BREF perform well in all three categories. This finding raises questions about the relevance and appropriateness of the former four tools for measuring and evaluating migrant wellbeing Table 4.

**Table 4:** Summary of COSMIN analysis

Tool Properties			Tools (year the tool developed)					
			SWLS (1985)	GHQ-12 (1988)	Ryff (1989)	WHO-5 (1998)	WHOQoL-BREF (1998)	PWI (2002)
Content validity	Asking patients	Relevance	Doubtful	Doubtful	Doubtful	Doubtful	Adequate	Adequate
		Comprehensiveness	Doubtful	Doubtful	Doubtful	Doubtful	Adequate	Adequate
		Comprehensibility	Doubtful	Doubtful	Doubtful	Doubtful	Adequate	Doubtful
	Asking experts	Relevance	not consulted	Doubtful	not consulted	Doubtful	Adequate	not consulted
		Comprehensiveness	not consulted	Doubtful	not consulted	Doubtful	Adequate	not consulted
Structural validity			Doubtful	Not applicable	Not applicable	Not applicable	Not applicable	Not applicable
Internal consistency			Very good	Very good	Very good	Very good	Very good	Very good
Cross-cultural validity			Inadequate	Doubtful	Inadequate	Doubtful	Very good	Adequate
Reliability			Inadequate	Inadequate	Doubtful	Doubtful	Very good	Very good
Measurement error			Doubtful	Adequate	Adequate	Adequate	Adequate	Very good
Criterion validity			Very good	Very good	Very good	Very good	Very good	Very good
Construct validity	Convergent validity		Very good	Very good	Very good	Adequate	Very good	Very good
	Known groups validity		Adequate	Very good	Very good	Adequate	Very good	Very good
Responsiveness	Comparison with gold standard		Inadequate	Very good	Very good	Very good	Very good	Very good
	Comparison with other instruments		Adequate	Very good	Adequate	Adequate	Doubtful	Very good
	Comparison between subgroups		Adequate	Very good	Adequate	Doubtful	Adequate	Very good
	Comparison before and after intervention		Not applicable	Not applicable	Not applicable	Not applicable	Not applicable	Not applicable

Source: authors own COSMIN evaluation

## Discussion

This systematic review investigates how the wellbeing of people who migrate from low- and/or middle-income countries to middle- and/or high-income countries has been conceptualized, defined, and measured.

Defining wellbeing is fundamental to measuring it, yet presents significant challenges as the concept is multidimensional [48]. Although 89(70.6%) articles did not explicitly define wellbeing, five distinct conceptualisations were identified, with subjective wellbeing the most common. In articles that did define wellbeing however, there were some inconsistencies between the stated definition of wellbeing and the measurement tool used. For example, five articles claiming to use a subjective wellbeing conceptualisation, used tools that define wellbeing using different constructs such as quality of life, mental health etc. (see Supplementary File Table 2).

Most tools identified in this review were used only once or twice, with few reported in three or more articles. Older tools tend to have been used more frequently, however frequency of use does not necessarily correlate with quality [33]. As shown, four of the older and more frequently used tools (SWLS, Ryff, WHO-5 and GHQ-12) did not have satisfactory scores according to the COSMIN analysis. It should be noted however, that the COSMIN checklist was developed in 2005, more recently than the evaluated tools. Using these evaluation criteria against older tools might have favoured the more recent tools while negatively impacting the older ones and may partially account for their unsatisfactory scores.

As the COSMIN evaluation indicated, PWI and WHOQoL-BREF are the tools with the fewest limitations for measuring migrant wellbeing. Until a new tool designed specifically for measuring migrant wellbeing is available, we recommend using these two tools for measuring migrants’ wellbeing. In addition to its high COSMIN score, PWI includes an optional question about spirituality/religion, which none of the other tools does [49]. Additionally, unlike all the other tools, PWI is regularly revised and updated.

Two of the most frequently used tools to measure migrant wellbeing (GHQ-12 and WHO-5) were created to screen and detect quite different constructs: psychiatric disorder and signs of depression, respectively. Nonetheless, 17 articles reported on the wellbeing of over 8,000 migrants based on these two tools (for studies that used these tools, see Supplementary File Table 1). While psychiatric disorders and depression are likely correlated with wellbeing, it should not be assumed that low/no psychiatric disorders/depression reflects high subjective wellbeing. This could result in misclassification which may lead to either no intervention or inappropriate intervention for those who do require support.

Only twenty-five articles reported using tools with validated translations for non-English versions (for the full list of studies that used translated/validated tools, see Supplementary File Table 3). More than half of the articles did not translate or did not indicate if tools were translated. Further, none of the tools used were specifically designed to measure migrant wellbeing.

When we evaluate the 126 articles included in this review through these four criteria, whether they have explicitly operationalized wellbeing in their study, whether they have used translated tools, whether the translated tools were validated and whether the conception of wellbeing in the articles and the tools used were aligned, only two articles [49, 50] meet these four criteria.

Many studies, for example [51, 52] report that migrants may have lower wellbeing than non-migrants. There are clearly factors unique to migrants, which may contribute to a lower level of wellbeing. It is also plausible that a portion of this difference can be attributed to cultural insensitivity of the tools used to measure the concept, or the cultural differences in ways questions are answered [54].

The six tools assessed using COSMIN in this review were designed and developed in the Global North, and validated in primarily Global North, non-migrant populations. It is likely that tools designed, developed and tested in white, middle-class populations, might not accurately measure the wellbeing of different groups.

Many scholars [18, 19, 21] have similarly argued that current wellbeing measurement tools developed in Global North and high-income contexts may overlook culturally significant elements essential to other regions and cultures. This study is the first however, to systematically review and evaluate the wellbeing tools used with migrant populations. Our findings cement these concerns, showing that the tools frequently used to measure migrant wellbeing have not been adequately developed to ensure appropriateness for those groups.

Our findings are relevant for researchers, practitioners, policymakers and migrants. For researchers, this review indicates that, beyond validation and translation, new culturally and contextually relevant wellbeing measurement tools are needed for measuring and tracking migrant wellbeing. Future research should prioritize development of new tools relevant to the realities of migrants including trauma, taking into account the diversity covered by the term migrant [23].

For practitioners, the review cautions that migrant wellbeing may not have been accurately measured creating barriers to understanding migrant wellbeing and designing appropriate interventions. For policy makers, the review calls into question the accuracy of the information on which they rely to make policy-based decisions. For migrants, improved measurement may lead to more appropriate evidence-based interventions and programs that can ultimately support their wellbeing while simultaneously deepening our understanding of difference, cultures and priorities.

### Strengths and Limitations

The strengths of this review include a systematic and comprehensive evidence synthesis and application of the COSMIN checklist to evaluate tool quality. It also has several limitations. Focusing only on studies conducted in English and in high- and middle-income countries, might have excluded tools and research published in other languages. Additionally, focusing only on the most used tools for the COSMIN analysis limits our ability to evaluate the applicability and relevance of the other less frequently used tools.

## Conclusion

There is currently no tool that has being specifically developed to measure migrant wellbeing. Most studies that measured migrant wellbeing failed to define the construct. Two of the most used tools were not designed to measure wellbeing and some studies that did define wellbeing, used tools not aligned with that definition. Most studies used tools that were designed for Global North populations. Through the COSMIN analysis, two tools identified as having the fewest limitations for the purpose of measuring migrant wellbeing (PWI and WHOQoL-BREF).

The findings of this review highlight the need for new and critical thinking about how to assess wellbeing in migrant populations. Without culturally appropriate tools, our understanding of migrant wellbeing will remain constrained, limiting our ability to develop effective interventions and policies. As migration continues to shape global demographics, the development of such tools is crucial to ensure equitable and effective public health interventions.

## Supplementary Information

Below is the link to the electronic supplementary material.

Supplementary Material 1

NEW Supplementary File Final Version [Submitted]

## Data Availability

Data is provided within the manuscript or supplementary information files.

## Footnotes

1 Covidence. 2024. Covidence. Available from: https://www.covidence.org/.
