# Supplementary material for: Have We Been Measuring Migrant Wellbeing all Wrong? Conceptualizing Migrant Wellbeing: A Systematic Review
Source: J Immigr Minor Health. 2025 Sep 10;28(2):463–75. doi: 10.1007/s10903-025-01773-z (PMC13083485; doi:10.1007/s10903-025-01773-z)
Supplement: Supplementary file 2 — NEW Supplementary File Final Version [Submitted] [file 10903_2025_1773_MOESM2_ESM.pdf]

Contents of the Supplementary File

Contents *of the Supplementary File*

Supplementary Table 1summary of the included studies 2

Supplementary Table 2: Wellbeing definitions in articles, compared with definitions on which the tools used are based 11

Supplementary Table 3: Studies that used translated, validated translations and aligned definitions 17

Supplementary Table 4: Articles/manuals/validation studies used for the COSMIN analysis 18

Supplementary Table 5: Subsequent validation studies 19

Supplementary material 1**:** Definitions of the COSMIN checklist terminologies 22

Supplementary Materials Reference List: 23

Supplementary Table 1summary of the included studies

(Ref)	Article ID	Country the study was conducted	Study design	Number of participants	Age group	Participant recruitment	Nationality/Ethnic/racial group of participants	Migrant's Generation	Type of migrants	Definition	Single/multi item tool	Number of tools	Name of the tool/s	Translation
[1]	Abad & Sheldon (2008).	USA	Cross-sectional	99	college aged'	Purposive	African, Asian, Spanish speaking and European/Canadian	2nd Generation	Refugees	No	Multi items	2	SHS & SWLS	Not reported
[2]	Abdo et al. (2019).	Jordan	Cross-sectional	1,583	18-75	Purposive	Syrian refugee and Poor Jordanian	1st Generation	Refugees	Yes	Multi items	1	WHOQOL-BREF	Not reported
[3]	Adedeji & Bullinger (2019).	Germany	Cross-sectional	518	19-56	Purposive	Sub-Saharan Africans	1st Generation	Refugees	Yes	Multi items	1	WHOQOL-BREF	Not reported
[4]	Adedeji et al (2021)	Germany	Cohort/longtudinal	518	19-56	Purposive	Sub-Saharan Africans	1st Generation	Refugees	Yes	Multi items	1	EUROHIS-QOL	reported
[5]	Adedeji et al. (2023).	Germany	Cross-sectional	518	19-56	Purposive	Sub-Saharan Africans	1st Generation	Refugees	No	Multi items	1	WHOQOL-BREF	reported
[6]	Al-Adhami et al. (2022).	Sweden	Cross-sectional	787	19-69	Convenience	Arabic speakers	1st Generation	Refugees	No	Multi items	1	GHQ-12	reported
[7]	Alexander et al. (2021).	Sweden	Cross-sectional	1,215	18-64	Random	Syrian	1st Generation	Refugees	No	Multi items	1	WHO-5	reported
[8]	Amit & Bar-lev (2015)	Israel	Cross-sectional	587	Mean 42.5	Random	(355 French immigrants and 232 FSU immigrants	1st Generation	Immigrants	Yes	Multi items	1	Adhoc scle	reported
[9]	Amit & Litwin (2010)	Israel	Cross-sectional	930	>=50	Existing survey	Asia, Africa, East Europe, West Europe America and FSU	2nd Generation	Immigrants	Yes	Single item	1	GLS	No
[10]	Anjara et al. (2017)	Singapor	Cross-sectional	182	18-50+	Purposive	Indonesians and Filipinos	1st Generation	Labour-migrants	Yes	Multi items	1	WHOQoL-Bref	No
[11]	Bernardo et al . (2022)	Macau (China)	Cross-sectional	249	20-59	Purposive	Fillipino	1st Generation	Labour-migrants	Yes	Multi items	1	SWLS	reported
[12]	Berry & Hou (2016).	Canada	Cross-sectional	7,003	>=15	Existing survey	From 182 source countries	1st Generation	Immigrants	No	Single item	1	GLS	reported
[13]	Berry & Hou (2019).	Canada	Cross-sectional	8,269	>=15	Existing survey	Immigrants and canadians	Mix	Immigrants	No	Single item	1	GLS	Not reported
[14]	Berry & Hou (2021)	Canada	Cross-sectional	21,282	>=15	Existing survey	Immigrants and canadians	Mix	Immigrants	No	Single item	1	GLS	Not reported
[15]	Blaauw & Pretorius (2023)	South Africa	Cross-sectional	104	<20->50	Snowball	SSA	1st Generation	Refugees	No	Single item	1	GLS	Not reported

(Ref)	Article ID	Country the study was conducted	Study design	Number of participants	Age group	Participant recruitment	Nationality/Ethnic/racial group of participants	Migrant's Generation	Type of migrants	Definition	Single/multi item tool	Number of tools	Name of the tool/s	Translation
[16]	Bobowik et al. (2011)	Spain	Cross-sectional	33048 (only1171 imm)	18-60	Existing survey	multiple	1st Generation	Immigrants	Yes	Single item	2	GLS (two)	Not reported
[17]	Bobowik et al. (2013).	Spain	Cross-sectional	1,250	18-64	Random	Multiple	1st Generation	Immigrants	No	Multi items	3	Ryff; GLS & SWS	Not reported
[18]	Buckingham & Suarez-Pedraza (2019).	USA	Cross-sectional	438	18-77	Snowball	Latin Americans	1st Generation	Labour-migrants	Yes	Multi items	1	I COPPE	reported
[19]	Burholt et al. (2018)	England & Wales	Cross-sectional	815	40-65+	Existing survey	Black African, Black Caribbean, Indian, Pakistani, Bangladeshi and Chinese	1st Generation	Immigrants	No	Single item	1	GLS	No
[20]	Burton-Jeangros et al. (2021)	Switzerland	Cross-sectional	601	19-74	Existing survey	not specified	1st Generation	Labour-migrants	No	Single item	1	GLS	Not reported
[21]	Carpentier & de la Sablonniere (2013).	Canada	Cross-sectional	120	17-52	Purposive	multiple	1st Generation	Immigrants	No	Multi items	1	SWLS	reported
[22]	Castaneda et al (2015)	Finland	Cross-sectional	1,795	18-64	Existing survey	Russian, Somali and Kurdish	1st Generation	Immigrants	No	Single item	1	GLS	Not reported
[23]	Cetin (2019)	Turkey	Cross-sectional	97	18-45	Random	53.6% Syria, 18.6 Egypt 15.5% Palestine 7.2% Oman and 5.2% Iraq	1st Generation	Refugees	Yes	Multi items	1	SWBS	reported
[24]	Chang (2015)	South Korea	Cross-sectional	64,972	mean 33.4	Existing survey	Korean-Chinese, Han Chinese, Vietnamese, Japanese, Filipinas, and Cambodian	1st Generation	Marriage Migrants	No	Single item	1	GLS	reported
[25]	Cheung et al. (2023)	China & The Netherlands	Cross-sectional	333(134 imm)	>=60	Purposive	Chiness	1st Generation	Immigrants	Yes	Multi items	1	WHOQoL-BREF	Not reported
[26]	Cleveland et al. (2023).	Canada & USA	Cross-sectional	203	18-50+	Purposive	74 % Chinese-Canadians, and 26 % were Indian-Americans	1st Generation	Labour-migrants	No	Multi items	2	SWLS & WHO-5	Not reported
[27]	Cobb et al. (2019).	USA	Cross-sectional	140	18-60	Purposive	Hispanic	1st Generation	Labour-migrants	Yes	Multi items	2	FS & SWLS	reported

(Ref)	Article ID	Country the study was conducted	Study design	Number of participants	Age group	Participant recruitment	Nationality/Ethnic/racial group of participants	Migrant's Generation	Type of migrants	Definition	Single/multi item tool	Number of tools	Name of the tool/s	Translation
[28]	Coffey et al. (2010).	Australia	Cross-sectional	17	28-57	Purposive	Afghanistan, Iraq, Iran or neighbouring Middle-Eastern countries	1st Generation	Refugees	No	Multi items	1	WHOQoL-Bref	reported
[29]	Cohen-Louck & Shechory-Bitton (2021)	Israel	Cross-sectional	341	18-50	Convenience	Ethiopians & FSU	1st Generation	Immigrants	No	Multi items	1	MHI	reported
[30]	Cramm & Nieboer (2018a)	The Netherlands	Cross-sectional	680	66-95	Purposive	Turkish	1st Generation	Immigrants	No	Multi items	1	SPF-ILs (Turkish version)	reported
[31]	Cramm & Nieboer (2018b)	The Netherlands	Cross-sectional	680	66-95	Purposive	Turkish	1st Generation	Immigrants	No	Multi items	1	SPF-ILs (Turkish version)	reported
[32]	Cross et al. (2008).	UK	Cross-sectional	605	18-64	Purposive	Multiple	1st Generation	Labour-migrants	No	Multi items	1	EuroQol EQ-5D	reported
[33]	Cuellar et al. (2004)	USA	Cross-sectional	353	45-88	Random	Mexican	Mix	Labour-migrants	Yes	Multi items	1	Index of Life Satisfaction	reported
[34]	Damri & Litwin (2016)	Israel	Cross-sectional	1,590	>=50	Existing survey	Veteran-Jews Arab-Israelis FSU immigrants	1st Generation	Immigrants	Yes	Multi items	1	CASP-19 scale	Not reported
[35]	D'isanto et al. (2016)	Italy	Cross-sectional	797	mean 36	Random	Africans, European, Asian and Americans	1st Generation	Illegal immigrants	No	Multi items	1	VAS	Not reported
[36]	Dolezal et al. (2021).	USA	Cross-sectional	112	18-44	Purposive	Muslim refugees	1st Generation	Refugees	No	Multi items	1	WHO-5	reported
[37]	Dou et al. (2022)	The Gallup World Poll data (2014 to 2019)	Cross-sectional	36,313	Mean 42	Existing survey	Multiple	1st Generation	Immigrants	No	Multi items	1	NEI & PEI	Not reported
[38]	Dryjanska & Zlotnick (2021).	USA	Cross-sectional	169	mean 45	Convenience	Hispanic/Latino	1st Generation	Immigrants	No	Multi items	1	SWLS	reported
[39]	Fassbender & Leyendecker (2018)	Germany	Cohort/longtudinal	327	24-59	Purposive	Turkish	Mix	Labour-migrants	No	Multi items	1	SWLS	Not reported
[40]	Garcia-cid et al. (2020).	Spain	Cross-sectional	1,714	16-74	Random	Mixed	1st Generation	Immigrants	No	Multi items	1	SWLS	reported
[41]	Goodkind (2005).	USA	Cohort/longtudinal	28	22-77	Purposive	Hmong	1st Generation	Refugees	No	Multi items	2	SLA & Rumbaut's PWS	reported
[42]	Haagsman et al. (2015)	The Netherlands	Cross-sectional	607	26-63	Purposive	Angolans and Nigerians	1st Generation	Labour-migrants	Yes	Single item	1	Adhoc scale	Not reported

(Ref)	Article ID	Country the study was conducted	Study design	Number of participants	Age group	Participant recruitment	Nationality/Ethnic/racial group of participants	Migrant's Generation	Type of migrants	Definition	Single/multi item tool	Number of tools	Name of the tool/s	Translation
[43]	Haj-Younes et al. (2020)	Norway	Cohort/longtudinal	353	median 34	Existing survey	Syrian	1st Generation	Refugees	No	Multi items	1	WHOQOL-BREF	reported
[44]	Hashemi et al. (2021).	Australia	Cross-sectional	382	20-39	Purposive	Middle Eastern	1st Generation	Refugees	Yes	Multi items	1	SWLS	No
[45]	Hendriks & Burger (2020)	EU15	Cross-sectional	7,044	not reported	Existing survey	Multiple	Mix	Immigrants	Yes	Single item	1	GLS	Not reported
[46]	Herrero et al. (2011).	Spain	Cross-sectional	350	18-71	Purposive	Latin American	1st Generation	Immigrants	Yes	Single item	2	GLS (two)	Not reported
[47]	Hsieh (2019)	USA	Cohort/longtudinal	142	61-79	Existing survey	Chiness Americans	1st Generation	Immigrants	No	Single item	1	GLS	Not reported
[48]	Htay et al. (2020)	Malaysia	Cross-sectional	192	mean 32.96	Convenience	Myanmar	1st Generation	Labour-migrants	No	Multi items	1	WHO-5	reported
[49]	Jackson et al. (2007)	USA	Cross-sectional	1,595	18-55+	Existing survey	Black Caribbean	Mix	Labour-migrants	No	Single item	1	GLS	No
[50]	Katz (2009)	Israel	Cross-sectional	212	65+	Purposive	Arab, kibbutzim & FSU	1st Generation	Immigrants	No	Multi items		Adhoc scale	Not reported
[51]	Kearns et al. (2017)	Scotland	Cross-sectional	7,191	16-40+	Existing survey	multiple	1st Generation	Refugees & Asylum seekers	No	Multi items	1	SF-12v2	Not reported
[52]	Khatiwada et al. (2021).	Japan	Cross-sectional	249	18-60	Convenience	Nepalese	1st Generation	Labour-migrants	No	Multi items	1	SWLS	reported
[53]	Klokgieters et al. (2019)	The Netherlands	Cohort/longtudinal	455	55-66	Purposive	Turkish and Moroccan immigrants	1st Generation	Immigrants	Yes	Multi items	1	CES-D	reported
[54]	Knies G. et al. (2016)	UK	Cohort/longtudinal	32,055	>=16	Existing survey	Multiple	Mix	Immigrants	No	Single item	1	GLS	No
[55]	Kuehne et al. (2015)	Germany	Cross-sectional	96(Quant) + 35(Qual)	20-64	Others	multiple	1st Generation	Undocumented migrants	No	Multi items	1	SF-12v2	reported
[56]	Kushnirovich & Youngmann (2017).	Israel	Cross-sectional	2,927	24-62	Existing survey	multiple	1st Generation	Immigrants	Yes	Multi items	2	Adhoc scale	Not reported
[57]	Lai et al. (2013)	Australia	Cross-sectional	2,151	not reported	Existing survey	Hong Kong Chinese; Chinese Australian; and Australian	Mix	Immigrants	No	Multi items	1	PWI	Not reported
[58]	Lai et al. (2019)	USA	Cross-sectional	2,717	>=60	Existing survey	Chinese	1st Generation	Immigrants	No	Single item	1	GLS	Not reported
[59]	Lats et al 2016).	USA	Cross-sectional	200	18-32	Convenience	Eastern European	1st Generation	Immigrants	No	Multi items	1	MHI	Not reported
[60]	Lee et al. (2022).	South Korea	Cross-sectional	11,188	20-59	Existing survey	East/Southeast/South Asia	1st Generation	Marriage Migrants	No	Single item	1	GLS	Not reported
[61]	Lee-Fong (2022)	USA	Cross-sectional	76	>=18	Purposive	multiple	1st Generation	Refugees	Yes	Multi items	1	SPWB	No

(Ref)	Article ID	Country the study was conducted	Study design	Number of participants	Age group	Participant recruitment	Nationality/Ethnic/racial group of participants	Migrant's Generation	Type of migrants	Definition	Single/multi item tool	Number of tools	Name of the tool/s	Translation
[62]	Leon-Pinilla et al. (2020).	Spain	Cross-sectional	186	18-62	Convenience	multiple	1st Generation	Asylum-seekers & Refugees	No	Multi items	1	SPANE	reported
[63]	Levy & Itzhaky (2016)	Israel	Cross-sectional	123	not reported	Convenience	Ethiopians	1st Generation	Immigrants	Yes	Multi items	1	Bradburn	reported
[64]	Lin et al. (2016).	Australia	Cross-sectional	119	>=65	Convenience	Chinese and Australian born elders	1st Generation	Immigrants	Yes	Multi items	1	WHOQoL-BREF	Not reported
[65]	Liu et al. (2021)	USA	Cross-sectional	3,157	>=60	Existing survey	Chinese	1st Generation	Immigrants	No	Single item	1	GLS	reported
[66]	Lofvander et al. (2014)	Sweden	Case-control	186	18-65	Purposive	Somalia, Iraqi and Swedish born	1st Generation	Immigrants	No	Multi items	1	WHOQoL-BREF	reported
[67]	Lonnqvist et al. (2015)	Finland	Cohort/longtudinal	225; 155; 133; 85 (sample at each cohort)	only mean age was reported	Purposive	Russian	1st Generation	Immigrants	No	Multi items	1	SWLS	reported
[68]	Madi et al. (2022).	Spain	Cross-sectional	1,250	18-64	Existing survey	Sub-Saharan Africa, Bolivia, Colombia, Morocco, and Romania	1st Generation	Immigrants	Yes	Multi items	2	PWS & SWS	Not reported
[69]	Maier et al. (2022).	Germany	Cross-sectional	744	18-67	Convenience	not specified	1st Generation	Refugees	No	Multi items	1	WHO-5	Not reported
[70]	Maki-opas et al. (2022)	Finland	Cohort/longtudinal	866	16-65+	Existing survey	not specified	1st Generation	Refugees	No	Multi items	1	WHOQoL-BREF	reported
[71]	Markovic et al. (2023)	Serbia	Cross-sectional	201	14-65	Convenience	multiple	1st Generation	Refugees	No	Multi items	1	WHO-5	reported
[72]	Martinez-Damia et al. (2023)	Italy	Cross-sectional	308	18-65+	Others	developing countries	1st Generation	Immigrants	Yes	Multi items	1	ICOPPE scale	reported
[73]	Martynowska et al. (2020).	UK	Cross-sectional	551	17-64	Purposive	Polish	1st Generation	Immigrants	No	Multi items	1	Ryff	reported
[74]	Matanov et al. (2013).	East & West Europe	Cross-sectional	3313 Balkan residents; 854 refugees = 4167	only mean age was reported : 42.3	Existing survey	Balkans	1st Generation	Refugees	No	Multi items	1	MANSA	Not reported
[75]	Merz & Consedine (2012).	USA	Cross-sectional	1,116	>=65	Existing survey	multiple	Mix	Immigrants	No	Multi items	1	Differential Emotions Scale	No
[76]	Miller et al. (2020)	Lebanon	Randomized control trial	151	not reported	Purposive	Syrian, Lebanese and Palestinian	1st Generation	Refugees	No	Multi items	1	WEMWBS	Not reported
[77]	Monteiro & Haan (2022)	Canada	Cross-sectional	6,842	15-75+	Existing survey	not specified. Check P.1404	Mix	Immigrants	No	Single item	1	GLS	Not reported

(Ref)	Article ID	Country the study was conducted	Study design	Number of participants	Age group	Participant recruitment	Nationality/Ethnic/racial group of participants	Migrant's Generation	Type of migrants	Definition	Single/multi item tool	Number of tools	Name of the tool/s	Translation
[78]	Morales et al. (2017)	Chile	Cross-sectional	431	>=18	Convenience	Peruvian and Colombian	1st Generation	Immigrants	Yes	Multi items	1	Keyes's Scale of Social Well-being	Not reported
[79]	Morville et al. (2014)	Denmark	Cross-sectional	43	20-50	Convenience	Afghanistan, Syria and Iran	1st Generation	Asylum-seekers	No	Multi items	1	WHO-5	Not reported
[80]	Morville et al. (2015)	Denmark	Cohort/longtudinal	17	20-50	Convenience	Afghanistan, Syria, and Iran	1st Generation	Asylum-seekers	No	Multi items	1	WHO-5	Not reported
[81]	Murphy & Mahalingam (2006).	USA	Cross-sectional	137	18-60	Purposive	Caribbean	1st Generation	Immigrants	No	Multi items	1	SWLS	No
[82]	Nielsen et al. (2023).	Denmark	Cross-sectional	90	13-56	Purposive	Syrian	1st Generation	Refugees	No	Multi items	1	SWEMWBS	reported
[83]	Nikendei et al. (2019).	Germany	Cross-sectional	228 at 1st contact; 88 at 2nd contact; 66 for therapy	18->=45	Convenience	not specified	1st Generation	Asylum-seekers	No	Multi items	1	WHO-5	reported
[84]	Pabilonia et al. (2010)	USA	Case-control	9	18-70	Purposive	Africa & South Asia	1st Generation	Asylum &Refugee	No	Multi items	1	WHOQoL-BREF	No
[85]	Paloma et al. (2014)	Spain	Cross-sectional	633	mean age reported , 31.9	Purposive	Moroccan	1st Generation	Immigrants	Yes	Multi items	1	SWLS	Not reported
[86]	Paparusso (2019)	Western Europe	Cross-sectional	7,468	>=15	Existing survey	Asia, Eastern Europe, Latin America, Middle East, North Africa, and Sub-Saharan Africa	1st Generation	Immigrants	No	Multi items	1	Adhoc scale	Not reported
[87]	Perera et al. (2022).	Colombia	Cross-sectional	72	>=18	Purposive	Venezuelan	1st Generation	Refugees	No	Multi items	2	WHO-5 & WHOQoL-BREF	reported
[88]	Plooy et al. (2019)	Australia	Cross-sectional	1,334	Onlu mean age reported , 46.15	Purposive	Anglo, Southern Asia, Confucian Asia and All other European	1st Generation	Immigrants	No	Multi items	1	MHC-SF	reported
[89]	Prapas & Mavreas (2019)	Greece	Cross-sectional	520	18-64	Purposive	Albanian immigrants, Pontic Greeks and native Greeks	1st Generation	Immigrants	Yes	Multi items	2	WHOQoL-BREF & SWLS	reported
[90]	Raffaelli et al. (2012)	USA	Cross-sectional	112	>=18	Purposive	Spanish-speaking Latina	1st Generation	Immigrants	No	Multi items	1	SWLS (Spanish version)	reported

(Ref)	Article ID	Country the study was conducted	Study design	Number of participants	Age group	Participant recruitment	Nationality/Ethnic/racial group of participants	Migrant's Generation	Type of migrants	Definition	Single/multi item tool	Number of tools	Name of the tool/s	Translation
[91]	Read-Wahidi & DeCaro (2017).	USA	Cross-sectional	60	19-60	Convenience	Mexican	1st Generation	Immigrants	No	Single item	1	GLS	Not reported
[92]	Regev & Slonim-Nevo (2019).	Israel	Cross-sectional	300	19-58	Snowball	Darfuri	1st Generation	Asylum-seekers	No	Multi items	1	WHOQoL-BREF	reported
[93]	Remennick (2005)	Israel	Cross-sectional	150	30-60	Purposive	former Soviet Union	1st Generation	Immigrants	No	NA		Adhoc scale	reported
[94]	Roy & Godfrey (2016)	USA	Cross-sectional	709	>=25	Existing survey	Dominican (N = 255), Puerto Rican (N = 242), and Mexican (N = 212)	1st Generation	Immigrants	Yes	Single item	3	GLS (three)	Not reported
[95]	Ryabichenko & Lebedeva (2016)	Russia	Cross-sectional	158	19-69	Snowball	Russians, Tajiks and Uzbeks	1st Generation	Immigrants	No	Multi items	1	SWLS	reported
[96]	Sander et al. (2019).	Denmark	Cross-sectional	646	Only mean age was reported 45.2	Purposive	Iraq, Afghanistan, Ex-Yugoslavia, Iran AND Lebanon	1st Generation	Refugees	No	Multi items	1	WHO-5	reported
[97]	Schiltz & Schiltz (2013)	Luxembourg	Cross-sectional	73	mean age: 37.19	Purposive	Africa & Europe	1st Generation	Refugees	No	Multi items	1	Index of Well-Being by Campbell	Not reported
[98]	Scottham & Dias (2010)	Japan	Cross-sectional	321	18-52	Purposive	Brazilians	1st Generation	Immigrants	No	Multi items	1	SWLS	reported
[99]	Simkin (2020)	Israel	Cross-sectional	204	18-80	Convenience	Multiple	1st Generation	Immigrants	No	Multi items	1	SWLS	reported
[100]	Stupar et al. (2014)	The Netherlands	Cross-sectional	1,236	16-86	Random	Turkey and Morocco (Non-western), Germany and Belgium (Western)	1st Generation	Immigrants	No	Multi items	1	SWLS	Not reported
[101]	Sulaiman-Hill & Thompson (2012).	Australia and New Zealand	Cross-sectional	81	<=30=>	Snowball	Afghan or Kurdish	1st Generation	Refugees	No	Multi items	1	PWI	Not reported
[102]	Taloyan et al. (2008)	Sweden	Cross-sectional	2,892	27-60	Existing survey	Kurdish and Swedish	1st Generation	Immigrants	No	Multi items	1	GHQ-12	Not reported
[103]	Tartakovsky & Walsh (2020).	Israel	Cross-sectional	400	18-75	Convenience	Former Soviet Union	1st Generation	Immigrants	No	Multi items	2	SWLS & PNES-UK	Not reported
[104]	Tinghog et al. (2009)	Sweden	Cross-sectional	720	20-75	Random	Finns, Iraqis and Iranians	1st Generation	Immigrants	Yes	Multi items	1	WHO-10	reported

(Ref)	Article ID	Country the study was conducted	Study design	Number of participants	Age group	Participant recruitment	Nationality/Ethnic/racial group of participants	Migrant's Generation	Type of migrants	Definition	Single/multi item tool	Number of tools	Name of the tool/s	Translation
[105]	Tip et al. (2019)	UK	Cohort/longtudinal	180	18-80	Convenience	Ethiopia, Iraq, Democratic Republic of Congo, and Somalia,	1st Generation	Refugees	No	Multi items	1	PNAS (Only 5 positive questions)	reported
[106]	Topal et al. (2012).	UK	Cross-sectional	416	17-65	Snowball	Turkish	1st Generation	Immigrants	No	Multi items	1	WHOQoL-BREF	reported
[107]	Toselli et al. (2018)	Italy	Cross-sectional	205	18-60	Purposive	North African ans Italian born	1st Generation	Immigrants	No	Multi items	2	Adhoc scale	Not reported
[108]	Urzua et al. (2017)	Chile	Cross-sectional	853	mean 33.2	Snowball	Colombian and Peruvian	1st Generation	Immigrants	No	Multi items	1	WHOQoL-BREF [A Spanish version]	reported
[109]	Urzua et al. (2018).	Chile	Cross-sectional	853	mean 33.2	Snowball	Peru, Colombia	1st Generation	Immigrants	No	Multi items	1	RPWS (Spanish version)	reported
[110]	Urzua et al. (2021a).	Chile	Cross-sectional	908	mean 36	Snowball	Colombian	1st Generation	Immigrants	Yes	Multi items	1	WHOQoL-BREF [A Spanish version]	reported
[111]	Urzua et al. (2021b).	Chile	Cross-sectional	962	18-89	Snowball	Colombian	1st Generation	Immigrants	Yes	Multi items	1	RPWS (Spanish version)	reported
[112]	Uskul & Greenglass (2005)	Canada	Cross-sectional	181	19-59	Convenience	Turkey	Mix	Immigrants	No	Multi items	1	LSS	reported
[113]	Uygun (2019)	Turkey	Cross-sectional	100	mean 33.8	Convenience	Syrian	1st Generation	Asylum-seekers	No	Multi items	2	WHO-5 & WHOQoL-BREF	reported
[114]	Uygun (2021)	Turkey	Case-control	207	18-65	Convenience	Syrian	1st Generation	Refugees	No	Multi items	1	WHO-5	Not reported
[115]	Var et al. (2013).	USA	Cross-sectional	100	18-70	Snowball	Asian	1st Generation	Refugees	Yes	Multi items	1	PWI	No
[116]	Villagrasa & Izquierdo (2018)	Spain	Cross-sectional	310	mean 36.1	Convenience	Latin America, Africa and Spanish	1st Generation	Labour-migrants	Yes	Multi items	1	GHQ-12	reported
[117]	Vohra & Adair (2000)	Canada	Cross-sectional	110	18-71	Purposive	Indians	1st Generation	Immigrants	Yes	Multi items	1	SWLS	No
[118]	Walther et al. (2020)	Germany	Cross-sectional	4,325	>=18	Existing survey	Syrian, Afghan, Iraqi, Eritrean, Other	1st Generation	Refugee & Asylum seekers	No	Single item	1	GLS	Not reported
[119]	Wangdahl et al. (2018)	Sweden	Cross-sectional	513	18->=45	Others	Afghanistan, Iraq, Iran, Somalia, and Syria	1st Generation	Refugees	No	Multi items	1	GHQ-12	reported

(Ref)	Article ID	Country the study was conducted	Study design	Number of participants	Age group	Participant recruitment	Nationality/Ethnic/racial group of participants	Migrant's Generation	Type of migrants	Definition	Single/multi item tool	Number of tools	Name of the tool/s	Translation
[120]	Xiao et al. (2020).	USA	Cross-sectional	223	>=18	Purposive	Far East, Southeast Asia, and/or the Indian subcontinent	Mix	Immigrants	Yes	Multi items	1	SF-36	No
[121]	Xu et al. (2022).	USA	Case-control	18	68-85	Purposive	Chinese	1st Generation	Immigrants	No	Multi items	1	LSIZ	Not reported
[122]	Yakhnich & Ben-Zur (2008)	Israel	Cross-sectional	301	not reported	Purposive	former Soviet Union	1st Generation	Immigrants	No	Multi items	1	SWLS	Not reported
[123]	Yalim (2021)	Turkey & USA	Cross-sectional	185	18-69	Snowball	Syrian	1st Generation	Refugees	No	Multi items	1	PWI	reported
[124]	Youngmann & Kushnirovich (2020).	Israel	Cross-sectional	3,272	mean 40.9	Existing survey	1261 Israeli Palestinians, 1127 FSU, 530 Europe & America, and 354 Africa	1st Generation	Immigrants	Yes	Multi items	1	Adhoc scale	Not reported
[125]	Zhang W. et al. (2020)	USA	Cross-sectional	430	>=55	Others	Chinese	Mix	Immigrants	No	Multi items	1	SWLS	Not reported
[126]	Zou et al. (2021)	China	Cross-sectional	928	>=18	Snowball	Sub-Saharan Africans	1st Generation	Immigrants	No	Multi items	1	WHOQoL-BREF	Not reported

Supplementary Table 2: Wellbeing definitions in articles, compared with definitions on which the tools used are based

(Ref)	Author/s	Article's definition of wellbeing	Approach of wellbeing (Article)	Approach of wellbeing (Tool)	Tool's definition of wellbeing	Aligned (Yes/No)
[2]	Abdo et al. (2019).	Quality of Life (QOL) can be defined as the general well-being of individuals and societies, outlining negative and positive features of life. It observes life satisfaction, including everything from physical health, family, education, employment, wealth, religious beliefs, finance, and the environment. P.2	QOL	WHOQOL-BREF	Quality of life is defined by the WHO as "individuals' perceptions of their position in life in the context of the culture and value systems in which they live and in relation to their goals, expectations, standards and concerns" (WHOQOL-BREF manual, P.4).	Yes
[3]	Adedeji & Bullinger (2019).	QoL is the evidence of 'individuals' perception of their position in life in the context of culture and value systems in which they live and in association with their goals, expectations, standards, and concerns. P.135	QOL	WHOQOL-BREF	Quality of life is defined by the WHO as "individuals' perceptions of their position in life in the context of the culture and value systems in which they live and in relation to their goals, expectations, standards and concerns" (WHOQOL-BREF manual, P.4).	Yes
[4]	Adedeji et al (2021)	QoL was conceptualised as a measure of the expression of the overall sense of life that includes aspects of health, environment, social relationships and addresses the diversity and multiculturalism that characterise today's society (Bullinger et al. 1995). It is presented as evidence of "individual's perception of their position in life in the context of the culture and value systems in which they live and in association with their goals, expectations, standards, and concerns" (WHO 1996).	QOL	EUROHIS-QOL 8-item Index*	Quality of life is defined by the WHO as "individuals' perceptions of their position in life in the context of the culture and value systems in which they live and in relation to their goals, expectations, standards and concerns" (WHOQOL-BREF manual, P.4).	Yes
[8]	Amit & Bar-lev (2015)	subjective well-being. It is defined as an overall assessment of an individual's quality of life according to his/her personal judgment and criteria. P.949	SWB	Own survey	subjective well-being. It is defined as an overall assessment of an individual's quality of life according to his/her personal judgment and criteria. P.949	Yes
[9]	Amit & Litwin (2010)	Life satisfaction is defined as an overall assessment of an individual's quality of life according to his or her personal judgment and criteria. P.90	LSF	GLS	No single definition is given	Yes
[10]	Anjara et al. (2017)	quality of life (defined as "an individual's perception of their position in life in the context of the culture and value systems in which they live and in relation to their goals, expectations, standards and concerns,". P.3	QOL	WHOQOL-BREF	Quality of life is defined by the WHO as "individuals' perceptions of their position in life in the context of the culture and value systems in which they live and in relation to their goals, expectations, standards and concerns" (WHOQOL-BREF manual, P.4).	Yes

(Ref)	Author/s	Article's definition of wellbeing	Approach of wellbeing (Article)	Approach of wellbeing (Tool)	Tool's definition of wellbeing	Aligned (Yes/No)
[11]	Bernardo et al . (2022)	well-being, which is a global assessment of an individual's satisfaction with life (Diener et al., 1985)	SWB	SWLS	Life satisfaction is a global assessment of a persons quality of life according to his chosen criteria. (Diener 1985, P.71)	Yes
[18]	Buckingham & Suarez-Pedraza (2019).	Wellbeing is a broad term that encompasses satisfaction with life as a whole, along with specific life domains	SWB	ICOPPE	Well-being is a positive state of affairs, as perceived by individual respondents (Prilleltensky et al., 2015, p.202).	Yes
[23]	Cetin (2019)	Existential well-being, on the other hand, is more about having a direction, purpose, and sense of meaning in life, being satisfied with life, and feeling optimistic about the future. P.65	SWB	Spiritual Well-Being Scale (SWBS)	The SWBS is a measure of one's perception of well-being understood in a holistic, spiritual (existential, religious, or both) sense (Paloutzian et al., 2021. p.415)	Yes
[25]	Cheung et al. (2023)	QoL by the WHO for the WHOQOL-BREF states "individuals' perceptions of their position in life in the context of the culture and value systems in which they live and in relation to their goals, expectations, standards and concerns". P.223	QOL	WHOQOL-BREF	Quality of life is defined by the WHO as "individuals' perceptions of their position in life in the context of the culture and value systems in which they live and in relation to their goals, expectations, standards and concerns" (WHOQOL-BREF manual, P.4).	Yes
[33]	Cuellar et al. (2004)	Well-being can be defined broadly to include physical health status or its perception, emotional well-being, and life satisfaction, including happiness. P.456	SWB	Index of Life Satisfaction	The Index of Life Satisfaction is a measure of the sum of 4 items: life satisfaction, satisfaction with house, satisfaction with neighborhood, and general health status (Cuellar et al. 2004, p.455)	Yes
[34]	Damri & Litwin (2016)	QOL, or positive well-being as it is also termed, is a comprehensive concept which reflects a range of complementary functional, emotional, interpersonal and material states that underlie health, happiness, comfort, and security in late life. P.300 the analysis employs the Higgs et al. (2003) and Hyde et al. (2003) paradigm of QOL which stems from need satisfaction theory. P.300	QOL	CASP-19 Scale	CASP-19 scale is composed of Control, Autonomy, Self-realization and Pleaseure and measures old age wellbeing in these foure areas (CASP-19 website).	Yes
[42]	Haagsman et al. (2015)	Subjective well-being generally refers to how people evaluate their lives and is a measure of their quality of life. P.2657	SWB	GLS	No single definition is given	Yes
[44]	Hashemi et al. (2021).	Subjective well-being is defined as people's overall evaluation of their lives, which comprises cognitive and affective components. P.111	SWB	SWLS	Life satisfaction is a global assessment of a persons quality of life according to his chosen criteria. (Diener 1985, P.71)	Yes

(Ref)	Author/s	Article's definition of wellbeing	Approach of wellbeing (Article)	Approach of wellbeing (Tool)	Tool's definition of wellbeing	Aligned (Yes/No)
[45]	Hendriks & Burger (2020)	Subjective well-being refers to the subjective enjoyment of one's life (Veenhoven 2012), which covers the extent to which an individual experiences both affectively pleasant feelings (i.e., an affective component) and perceives oneself as obtaining what one wants from life (i.e., a cognitive component). P.1986	SWB	GLS	No single definition is given	Yes
[53]	Klokgieters et al. (2019)	Well-being is defined as a person's evaluation and judgment of his or her quality of life as a whole. P. 627	SWB	CES-D	The Centre for Epidemiologic Studies Depression Scale (CES-D Scale) was developed for using studies of the epidemiology of depressive symptomatology in the general population (Radloff, 1977, P. 385).	No
[61]	Lee-Fong (2022)	Psychological wellbeing is a state of optimal mental and emotional functioning that is based upon six facets of wellness: autonomy, environmental mastery, self-acceptance, personal growth, a sense of purpose in life, and positive relationships. P.402	PWB	Ryff's PWS	Wellbeing is a function of these six domains: self-acceptance, positive relations with others, autonomy, environmental mastery, purpose in life, and personal growth. (Ryff, 1989a, P. 1072).	Yes
[63]	Levy & Itzhaky (2016)	well-being, defined as an emotional, subjective feeling that indicates the extent of the individual's enjoyment, happiness, and satisfaction with life. P. 2	SWB	Bradburn (1969)	The model of psychological well-being is as a function of two independent dimensions- positive and negative affect, each of which is related to wellbeing by an independent set of variables (Bradburn 1969, p.13)	Yes
[64]	Lin et al. (2016).	Psychological well-being is the individual realizes his or her own abilities, can cope with the normal stresses of life, can work productively and fruitfully, and is able to make a contribution to his or her community. P.1672	PWB	WHOQOL-BREF	Quality of life is defined by the WHO as "individuals' perceptions of their position in life in the context of the culture and value systems in which they live and in relation to their goals, expectations, standards and concerns" (WHOQOL-BREF manual, P.4).	No
[72]	Martinez-Damia et al. (2023)	we define subjective wellbeing as the satisfaction of needs across different domains, namely the personal sphere, relationships with relevant people in one's life as well as relationships with the community of residence, main occupation, overall health, emotional life, and financial situation. P.382	SWB	ICOPPE	Well-being is a positive state of affairs, as perceived by individual respondents (Prilleltensky et al., 2015, p.202).	Yes
[78]	Morales et al. (2017)	Social well-being, defined as 'the appraisal of one's circumstance and functioning in society' [1; p. 122; 19]. To achieve the objective measurement of social well-being, Keyes developed an instrument called the Scale of Social Well-being which measures each of the five proposed theoretical dimensions: Social Integration, Social Acceptance, Social	SOWB	Scale of Social Well-being	The Social Well-Being Scale (Keyes, 1998) was created to explore and measure the nature of a well-lived life. The Social Well-Being Scale is based on five thematic nuclei: social integration, social acceptance, social contribution, social actualization and social coherence (Keyes, 1998).	Yes

(Ref)	Author/s	Article's definition of wellbeing	Approach of wellbeing (Article)	Approach of wellbeing (Tool)	Tool's definition of wellbeing	Aligned (Yes/No)
		Contribution, Social Actualisation, and Social Coherence. P.1142				
[85]	Paloma et al. (2014)	This report defines well-being as the life satisfaction as expressed by an individual and assumes that it is a multilevel and value-dependent phenomenon, strongly related with access to resources, and therefore conditioned by norms and values of social justice in the receiving society. P.1	SWB	SWLS	Life satisfaction is a global assessment of a persons quality of life according to his chosen criteria. (Diener 1985, P.71)	Yes
[104]	Tinghog et al. (2009)	commonly proposed definition of SWB as 'a person's cognitive and affective appraisal of his or her life'. P.77	SWB	WHO (10) Well-Being Index	Measures mental wellbeing.	No
[110]	Urzua et al. (2021a).	level of satisfaction and well-being in several domains of daily life [4] that is the product of a cognitive evaluation process based on subjective standards of comparison. P.1	SWB	WHOQOL-BREF	Quality of life is defined by the WHO as "individuals' perceptions of their position in life in the context of the culture and value systems in which they live and in relation to their goals, expectations, standards and concerns" (WHOQOL-BREF manual, P.4).	No
[111]	Urzua et al. (2021b).	Psychological well-being is understood as a state of harmony and psychological fullness, where growth and personal development are the main factors of positive functioning. P. 2	PWB	Ryff's PWS	Wellbeing is a function of these six domians: self-acceptance, positive relations with others, authonomy, environmental mastry, purpose in life, and personal growth. (Ryff, 1989a, P. 1072).	Yes
[115]	Var et al. (2013).	well-being is primarily related to "what people feel they are able to do," while satisfaction with life is more associated to "what they actually have or perceived to be by the rest of society". P.80	SWB	PWI	Subjective wellbeing (SWB) is a broad term, referring to a person's overall sense of wellbeing, happiness, and life satisfaction. (PWI, 2024. P.2)	Yes
[116]	Villagrasa & Izquierdo (2018)	Well-being could be seen as an individual perception of satisfaction and fulfillment in all life domains, including work. P.4	SWB	GHQ-12	The tool was not designed/intended for measuring wellbeing and hence no definition of wellbeing provided.	No

(Ref)	Author/s	Article's definition of wellbeing	Approach of wellbeing (Article)	Approach of wellbeing (Tool)	Tool's definition of wellbeing	Aligned (Yes/No)
[117]	Vohra & Adair (2000)	life satisfaction rather than economic factors or happiness were used as indicators of well-being in this study.... Life satisfaction is defined as a 'global assessment of a person's quality of life according to his(/her) chosen criteria; (Shin & Johnson, 1978, p. 478). Satisfaction of life depends not only on what people have, but on a criteria that people choose themselves and how they judge their own achievements (Diener et al., 1985). P.111	LSF	SWLS	Life satisfaction is a global assessment of a persons quality of life according to his chosen criteria. (Diener 1985, P.71)	Yes
[120]	Xiao et al. (2020).	QOL is"individuals' perception of their position in life in the physical, emotional, social, mental, spiritual and other areas". P.713	QOL	SF-36 (adopted)	Quality of life (QOL) was adapted from 36-Item Short Form Survey Instrument (SF-36) (Lins & Carvalho, 2016), including areas of physical and emotional health problems, mobility, and bodily pain. Table 3 presents detailed items of these areas (except bodily pain). Xiao et al., 2020).	Yes
[124]	Youngmann & Kushnirovich (2020).	well-being is defined as emotional well-being (EWB), namely, the emotional quality of an individual's everyday experience; the frequency and intensity of experiences of joy, stress, sadness, anger, and affection that make one's life pleasant or unpleasant. P.2	SWB	GLS	No single definition is given	Yes
[16]	Bobowik et al. (2011)	it can be defined as an optimal psychological functioning andexperience which favours both a positive hedonic state and the development of skills and personal growth. P.402	SWB	GLS	No single definition is given	Yes
[27]	Cobb et al. (2019).	Psychological well-being is defined as a positive construct that includes feeling competent, purpose in life, self-acceptance, ability to meet the demands of one's social environment, and self-determined decision making. P.281	PWB	Flourishing Scale	The Flourishing Scale is a brief 8-item summary measure of the respondent's self-perceived success in important areas such as relationships, self-esteem, purpose, and optimism (Diener et al., 2010, p. 143).	Yes
				SWLS	SWLS: Life satisfaction is a global assessment of a persons quality of life according to his chosen criteria. (Diener 1985, P.71)	No
[46]	Herrero et al. (2011).	For Diener et al. (1999), SWB is a ''broad category of phenomena that includes people's emotional responses, domain satisfactions, and global judgements of life satisfaction'' (p. 277). In other words, SWB refers to the cognitive and affective evaluations of one's own life. P. 761-762	SWB	GLS	No single definition is given	Yes

(Ref)	Author/s	Article's definition of wellbeing	Approach of wellbeing (Article)	Approach of wellbeing (Tool)	Tool's definition of wellbeing	Aligned (Yes/No)
[56]	Kushnirovich & Youngmann (2017).	psychological well-being, is defined as a combination of feeling good and functioning well. P.52	PWB	GLS	No single definition is given	Yes
[68]	Madi et al. (2022).	Psychological wellbeing is the integration of positive psychological functioning including self-actualization, individuation, and perceptions of personal maturity. P.76	PWB	Ryff's PWS	Ryff's: Wellbeing is a function of these six domains: self-acceptance, positive relations with others, autonomy, environmental mastery, purpose in life, and personal growth. (Ryff, 1989a, P. 1072).	Yes
				Keyes's Social wellbeing scale	The Social Well-Being Scale (Keyes, 1998) was created to explore and measure the nature of a well-lived life. The Social Well-Being Scale is based on five thematic nuclei: social integration, social acceptance, social contribution, social actualization and social coherence (Keyes, 1998).	No
[89]	Prapas & Mavreas (2019)	Subjective well-being reflects a relative stable feeling of happiness one has towards his or her life, reflecting cognitive component as a general perceived life satisfaction. P. 78	SWB	WHOQOL-BREF	WHOQOL-BREF: Quality of life is defined by the WHO as "individuals' perceptions of their position in life in the context of the culture and value systems in which they live and in relation to their goals, expectations, standards and concerns". (WHOQOL-BREF manual, P.4).	No
				SWLS	SWLS: Life satisfaction is a global assessment of a persons quality of life according to his chosen criteria. (Diener 1985, P.71)	Yes
[94]	Roy & Godfrey (2016)	Subjective well-being refers to an individual's appraisal of one's life circumstances using both affective and objective information […] and is commonly evaluated using measures of global and/or domain-specific satisfaction. P861	SWB	GLS	No single definition is given	Yes

Supplementary Table 3: Studies that used translated, validated translations and aligned definitions

(Ref)	Articles	Name of the used tool/s	Translated	Validated	Aligned
[4]	Adedeji et al (2021)	EUROHIS-QOL 8-item Index	√		N*
[5]	Adedeji et al. (2023).	WHOQoL-BREF	√		Y
[6]	Al-Adhami et al. (2022).	GHQ-12	√	√	N
[7]	Alexander et al. (2021).	WHO-5	√	√	N
[8]	Amit & Bar-lev (2015)	Adhoc scale	√		Y
[11]	Bernardo et al . (2022)	SWLS	√		Y
[12]	Berry & Hou (2016).	GLS	√		N
[18]	Buckingham & Suarez-Pedraza (2019).	ICOPPE scale	√		Y
[21]	Carpentier & de la Sablonniere (2013).	SWLS	√		N
[23]	Cetin (2019)	SWBS	√		Y
[24]	Chang (2015)	GLS	√		N
[27]	**Cobb et al. (2019).**	**SWLS & FS****	√	√	**Y**
[28]	Coffey et al. (2010).	WHOQoL-BREF	√	√	N
[29]	Cohen-Louck & Shechory-Bitton (2021)	MHI	√	√	N
[30]	Cramm & Nieboer (2018a)	SPF-ILs (Turkish version)	√	√	N
[31]	Cramm & Nieboer (2018b)	SPF-ILs (Turkish version)	√	√	N
[32]	Cross et al. (2008).	EuroQol EQ-5D	√		N
[33]	Cuellar et al. (2004)	Index of Life Satisfaction	√		Y
[36]	Dolezal et al. (2021).	WHO-5	√	√	N
[38]	Dryjanska & Zlotnick (2021).	SWLS	√		N
[40]	Garcia-cid et al. (2020).	SWLS	√		N
[41]	Goodkind (2005).	Rumbaut's PWS & SLA	√		N
[43]	Haj-Younes et al. (2020)	WHOQoL-BREF	√	√	N
[48]	Htay et al. (2020)	WHO-5	√		N
[52]	Khatiwada et al. (2021).	SWLS	√	√	N
[53]	Klokgieters et al. (2019)	CES-D	√		N
[55]	Kuehne et al. (2015)	SF-12v2	√	√	N
[62]	Leon-Pinilla et al. (2020).	SPANE	√		N
[63]	Levy & Itzhaky (2016)	Bradburn (1969)	√		Y
[65]	Liu et al. (2021)	GLS	√		N
[66]	Lofvander et al. (2014)	WHOQoL-BREF	√		N
[67]	Lonnqvist et al. (2015)	SWLS	√		N
[70]	Maki-opas et al. (2022)	WHOQoL-BREF	√	√	N
[71]	Markovic et al. (2023)	WHO-5	√		N
[72]	**Martinez-Damia et al. (2023)**	**ICOPPE scale**	√	√	**Y**
[73]	Martynowska et al. (2020).	Ryff	√		N
[82]	Nielsen et al. (2023).	SWEMWBS	√		N
[83]	Nikendei et al. (2019).	WHO-5	√	√	N
[87]	Perera et al. (2022).	WHOQoL-BREF & WHO-5	√	√	N
[88]	Plooy et al. (2019)	MHC-SF	√		N
[89]	Prapas & Mavreas (2019)	WHOQoL-BREF & SWLS	√	√	N
[90]	Raffaelli et al. (2012)	SWLS	√	√	N
[92]	Regev & Slonim-Nevo (2019).	WHOQoL-BREF	√		N
[93]	Remennick (2005)	Adhoc scale	√		N
[95]	Ryabichenko & Lebedeva (2016)	SWLS	√		N
[96]	Sander et al. (2019).	WHO-5	√	√	N
[98]	Scottham & Dias (2010)	SWLS	√		N
[99]	Simkin (2020)	SWLS	√	√	N
[104]	Tinghog et al. (2009)	WHO-10	√		N
[105]	Tip et al. (2019)	PNAS (Only 5 positive questions)	√		N
[106]	Topal et al. (2012).	WHOQoL-BREF	√	√	N
[108]	Urzua et al. (2017)	WHOQoL-BREF	√	√	N
[109]	Urzua et al. (2018).	Ryff	√		N
[110]	Urzua et al. (2021a).	WHOQoL-BREF	√	√	N
[111]	Urzua et al. (2021b).	Ryff	√		Y
[112]	Uskul & Greenglass (2005)	LSS	√		N
[114]	Uygun (2019)	WHOQoL-BREF & WHO-5	√	√	N
[116]	Villagrasa & Izquierdo (2018)	GHQ-12	√	√	N

[119]	Wangdahl et al. (2018)	GHQ-12	√		N
[123]	Yalim (2021)	PWI	√	√	N

**only FS (Flourishing Scale) is aligned.

Supplementary Table 4: Articles/manuals/validation studies used for the COSMIN analysis

(Ref)	Articles/manuals/ validation studies used for COSMIN Analysis	Tool
[127]	Cummins et al. (2003)	PWI
[128]	International Wellbeing Group (2013)	PWI
[129]	Diener et al. (1985)	SWLS
[130]	Goldberg and Williams (1991)	GHQ
[131]	Ryff (1989)	Ryff
[132]	Ryff and Kayes (1995)	Ryff
[133]	The WHOQOL Group (1994)	WHOQOL-BREF
[134]	The WHOQOL Group (1994)	WHOQOL-BREF
[135]	The WHOQOL Group (1995)	WHOQOL-BREF
[136]	WHO (1998)	WHOQOL-BREF
[137]	Topp et al. (2015)	WHO-5
[138]	WHO-EURO (1998)	WHO-5

Supplementary Table 5: Subsequent validation studies

	Author	DOI	Tool	Validation language	Country	Remark
1	Esteban et al (2023)	https://doi.org/10.1016/j.heliyon.2023.e16843	WHO-5	Spanish	Peruvian South	
2	Latifa et al (2023)	https://doi.org/10.15408/jp3i.v12i2.33964	WHO-5	Indonesia	Indonesia	
3	Pheko et al (2023)	https://doi.org/10.1177/21582440231198387	WHO-5	Multiple	Botswana, Zimbabwe, Malaysia, and possibly, other similar settings	Multiple country study
4	Quansah et al (2022)	https://doi.org/10.3390/children9070991	WHO-5	Not specified	Ghana	The inclusion criteria is being able to read and write English language
5	Kassab (2023)	https://doi.org/10.1007/s10862-023-10027-x	WHO-5	Arabic	Saudi	
6	Suhaimi et al (2022)	https://doi.org/10.3390/ijerph19074415	WHO-5	Malaya	Malaysia	
7	Perera et al (2020)	https://doi.org/10.1186/s12955-020-01532-8	WHO-5	Sinhala	Sri Lanka	
8	Cichon et al (2020)	https://doi.org/10.1016/j.diabres.2019.107970	WHO-5	Polish	Poland	
9	Du et al (2023)	https://doi.org/10.1186/s12888-023-05381-9	WHO-5	Chinese	China	
10	Faruk et al (2021)	https://doi.org/10.1017/gmh.2021.26	WHO-5	Bangla	Bangladeshi	
11	Sibai et al (2009)	https://doi.org/10.1002/gps.2079	WHO-5	Arabic	Lebanon	
12	Sischka et al (2025)	https://doi.org/10.1177/10731911241309452	WHO-5	Multiple	Multiple (43 countries)	included European, Central Asia, and North American countries
13	Akin (2008)	https://eric.ed.gov/?id=ej837765	Ryff	Turkish	Turkey	
14	Ansari (2010)	NA	Ryff	Urdu	Canada	Ansari, S. A. (2010). Cross validation of Ryff scales of psychological wellbeing: Translation into Urdu language. Pakistan Business Review, 12(2), 244-259
15	Diaz et al (2006)	PMID: 17296089	Ryff	Spanish	Spain	The article is in Spanish, only abstract in English
16	Avsec & Sočan (2010)	https://doi.org/10.20419/2009.18.286	Ryff	Slovene	Slovene	The article is in Slovene, only abstract in English
17	González et al (2023)	http://ref.scielo.org/4xpfdr	Ryff	Spanish	Cuba	The article is in Spanish, only abstract in English

18	Jibeen & Khalid (2012)	NA	Ryff	Urdu	Canada (Pakistani immigrants)	Jibeen, T., & Khalid, R. (2012). Cross validation of Ryff's scales of psychological well-being: Translation into Urdu language. The International Journal of Educational and Psychological Assessment, 10(2), 67-91
19	Ruini et al (2021)	NA	Ryff	Italian	Italy	Ruini, C., Ottolini, F., Rafanelli, C., Ryff, C., & Fava, G. A. (2003). La validazione italiana delle psychological well-being scales (PWB) [Italian validation of psychological well-being scales (PWB)]. Rivista di Psichiatria, 38(3), 117-130
20	Saajanaho et al (2020)	https://doi.org/10.1080/13607863.2020.1725801	Ryff	Finnish	Finland	
21	Sasaki et al (2020)	https://doi.org/10.1186/s40359-020-00441-1	Ryff	Japanese	Japan	
22	Stavraki et al (2022)	https://doi.org/10.7334/psicothema2021.235	Ryff	Spanish	Spain	
23	Triadó et al (2007)	https://doi.org/10.2466/pr0.100.4.1151-1164	Ryff	Spanish	Spain	
24	John et al (2013)	https://doi.org/10.12968/bjcn.2006.11.9.21760	GHQ-12	Tamil	India	
25	Montazeri et al (2003)	https://doi.org/10.1186/1477-7525-1-66	GHQ-12	Persian	Iran	
26	El-Rufaie & Daradkeh (1996)	https://doi.org/10.1192/bjp.169.5.662	GHQ-12 & GHQQ-30	Arabic	UAE	
27	Kashyap et al (2017)	https://doi.org/10.1186/s12888-017-1253-y	GHQ-12	Hindu	India	
28	Balajti et al (2007)	https://doi.org/10.1556/mental.8.2007.2.4	GHQ-12	Hungarian	Hungary	Article in Hungarian
29	Benoni et al (2024)	https://doi.org/10.1186/s12955-024-02226-1	GHQ-12	Ukrainian	Italy	
30	Chavez-Espinoza et al (2023)	https://doi.org/10.17533/udea.iatreia.177	GHQ-12	Peruvian	Peru	Article in Spanish
31	Schrnitz et al (1999)	https://doi.org/10.1111/j.1600-0447.1999.tb10898.x	GHQ-12	German	German	Only found abstract
32	Abdallah (1998)	https://doi.org/10.1080/02673843.1998.9747816	SWLS	Arabic	West Bank	
33	Nooripour et al (2023)	https://doi.org/10.1007/s12144-021-01662-2	SWLS	Persian	Iran	
34	Lyrakos et al (2013)	doi:10.1016/S0924-9338(13)76471-X	SWLS	Greek	Greek	
35	Blais et al (1989)	NA	SWLS	French-Canadian	Canada	The article is in French , only abstract in English
36	Laranjeira (2009)	https://doi.org/10.1080/13548500802459900	SWLS	Portuguese	Portugal	
37	Lucas-Carrasco et al (2013)	https://doi.org/10.3109/09638288.2013.825650	SWLS	Spanish	Spain	
38	Bagherzadeh et al (2018)	doi:10.1017/sjp.2018.2	SWLS	Spanish	Chile	

39	Jaiswal et al (2020)	NA	SWLS	Hindi	India	Jaiswal, A., Singh, N., Rani, R., Sarraf, S., Pandey, D., & Pandey, V. (2020). Standardization and validation of Hindi version of satisfaction with life scale. Indian J Psychol Educ, 10, 106-11.
40	Berríos-Riquelme et al (2021)	https://doi.org/10.4067/s0718-48082021000200199	SWLS	Latinos immigrants	Latinos immigrants in Chile, Spain, and USA	Article in Spanish, only abstract in English

Supplementary material 1: Definitions of the COSMIN checklist terminologies

Definitions of the COSMIN checklist terminologies. Taken verbatim from [139, p. 743]:

- Reliability: The degree to which the measurement is free from measurement error.
- Internal consistency: The degree of the interrelatedness among the items.
- Measurement error: The systematic and random error of a patient's score that is not attributed to true changes in the construct to be measured.
- Content validity: The degree to which the content of an HR-PRO instrument is an adequate reflection of the construct to be measured.
- Construct validity: The degree to which the scores of an HR-PRO instrument are consistent with hypotheses (for instance with regard to internal relationships, relationships to scores of other instruments, or differences between relevant groups) based on the assumption that the HR-PRO instrument validly measures the construct to be measured.
- Structural validity: The degree to which the scores of an HR-PRO instrument are an adequate reflection of the dimensionality of the construct to be measured.
- Cross-cultural validity: The degree to which the performance of the items on a translated or culturally adapted HR-PRO instrument are an adequate reflection of the performance of the items of the original version of the HR-PRO instrument.
- Criterion validity: The degree to which the scores of an HR-PRO instrument are an adequate reflection of a ‘‘gold standard’’.
- Responsiveness: The ability of an HR-PRO instrument to detect change over time in the construct to be measured.

Supplementary Materials Reference List:

[1] N. S. Abad and K. M. Sheldon, 'Parental autonomy support and ethnic culture identification among second-generation immigrants.', *Journal of Family Psychology*, vol. 22, no. 4, pp. 652–657, Aug. 2008, doi: 10.1037/0893-3200.22.3.652.

[2] N. Abdo, F. Sweidan, and A. Batieha, 'Quality-of-life among Syrian refugees residing outside camps in Jordan relative to Jordanians and other countries', *PeerJ*, vol. 7, p. e6454, Mar. 2019, doi: 10.7717/peerj.6454.

[3] A. Adedeji and M. Bullinger, 'Subjective integration and quality of life of Sub-Saharan African migrants in Germany', *Public Health*, vol. 174, pp. 134–144, Sep. 2019, doi: 10.1016/j.puhe.2019.05.031.

[4] A. Adedeji, N. Silva, and M. Bullinger, 'Cognitive and Structural Social Capital as Predictors of Quality of Life for Sub-Saharan African Migrants in Germany', *Applied Research Quality Life*, vol. 16, no. 3, pp. 1003–1017, Jun. 2021, doi: 10.1007/s11482-019-09784-3.

[5] A. Adedeji, T. Y. Akintunde, F. Metzner, and E. Idemudia, 'Psychological health outcome of Sub-Saharan African migrants in Germany', *ELECTRON J GEN MED*, vol. 20, no. 1, p. em428, Jan. 2023, doi: 10.29333/ejgm/12602.

[6] M. Al-Adhami, E. Berglund, J. Wångdahl, and R. Salari, 'A cross-sectional study of health and well-being among newly settled refugee migrants in Sweden–The role of health literacy, social support and self-efficacy', *PLoS ONE*, vol. 17, no. 12, p. e0279397, Dec. 2022, doi: 10.1371/journal.pone.0279397.

[7] N. Alexander, S. Mathilde, and S. Øivind, 'Post-migration Stressors and Subjective Well-Being in Adult Syrian Refugees Resettled in Sweden: A Gender Perspective', *Front. Public Health*, vol. 9, p. 717353, Sep. 2021, doi: 10.3389/fpubh.2021.717353.

[8] K. Amit and S. Bar-Lev, 'Immigrants' Sense of Belonging to the Host Country: The Role of Life Satisfaction, Language Proficiency, and Religious Motives', *Soc Indic Res*, vol. 124, no. 3, pp. 947–961, Dec. 2015, doi: 10.1007/s11205-014-0823-3.

[9] K. Amit and H. Litwin, 'The Subjective Well-Being of Immigrants Aged 50 and Older in Israel.', *Social indicators research*, vol. 98, no. 1, pp. 89–104, 2010.

[10] S. G. Anjara, L. B. Nellums, C. Bonetto, and T. Van Bortel, 'Stress, health and quality of life of female migrant domestic workers in Singapore: a cross-sectional study', *BMC Women's Health*, vol. 17, no. 1, p. 98, Dec. 2017, doi: 10.1186/s12905-017-0442-7.

[11] A. B. I. Bernardo, M. A. Daganzo, and J. F. C. Burgos, 'Heritage Culture Detachment Predicts Hope and Well-Being of Filipino Migrant Workers: A Study of Low- and Semi-skilled Filipino Workers in Macau', *Psychol Stud*, vol. 67, no. 3, pp. 372–384, Sep. 2022, doi: 10.1007/s12646-022-00661-y.

[12] J. W. Berry and F. Hou, 'Immigrant acculturation and wellbeing in Canada.', *Canadian Psychology / Psychologie canadienne*, vol. 57, no. 4, pp. 254–264, Nov. 2016, doi: 10.1037/cap0000064.

[13] J. W. Berry and F. Hou, 'Multiple belongings and psychological well-being among immigrants and the second generation in Canada.', *Canadian Journal of Behavioural Science / Revue canadienne des sciences du comportement*, vol. 51, no. 3, pp. 159–170, Jul. 2019, doi: 10.1037/cbs0000130.

[14] J. W. Berry and F. Hou, 'Immigrant acculturation and wellbeing across generations and settlement contexts in Canada', *International Review of Psychiatry*, vol. 33, no. 1–2, pp. 140–153, Feb. 2021, doi: 10.1080/09540261.2020.1750801.

[15] P. F. Blaauw and M. Pretorius, '"I am 30 and I have nothing": the context of reception and the lived experiences of foreign migrants working as car guards in Johannesburg's

West Rand', *GeoJournal*, vol. 88, no. 1, pp. 121–135, Feb. 2022, doi: 10.1007/s10708-022-10594-8.

[16] M. Bobowik, N. Basabe, D. Páez, A. Jiménez, and M. Á. Bilbao, 'Personal Values and Well-Being among Europeans, Spanish Natives and Immigrants to Spain: Does the Culture Matter?', *J Happiness Stud*, vol. 12, no. 3, pp. 401–419, Jun. 2011, doi: 10.1007/s10902-010-9202-1.

[17] M. Bobowik, N. Basabe, and D. Páez, 'The bright side of migration: Hedonic, psychological, and social well-being in immigrants in Spain', *Social Science Research*, vol. 51, pp. 189–204, May 2015, doi: 10.1016/j.ssresearch.2014.09.011.

[18] S. L. Buckingham and M. C. Suarez-Pedraza, '"It has cost me a lot to adapt to here": The divergence of real acculturation from ideal acculturation impacts Latinx immigrants' psychosocial wellbeing.', *American Journal of Orthopsychiatry*, vol. 89, no. 4, pp. 406–419, 2019, doi: 10.1037/ort0000329.

[19] V. Burholt, C. Dobbs, and C. Victor, 'Social support networks of older migrants in England and Wales: the role of collectivist culture', *Ageing and Society*, vol. 38, no. 7, pp. 1453–1477, Jul. 2018, doi: 10.1017/S0144686X17000034.

[20] C. Burton-Jeangros, A. Duvoisin, L. Consoli, J. Fakhoury, and Y. Jackson, 'The well-being of newly regularized migrant workers: Determinants of their satisfaction with life as compared to undocumented migrant workers and regular local residents', *CMS*, vol. 9, no. 1, p. 42, Dec. 2021, doi: 10.1186/s40878-021-00244-2.

[21] J. Carpentier and R. De La Sablonnière, 'Identity Profiles and Well-Being of Multicultural Immigrants: The Case of Canadian Immigrants Living in Quebec', *Front. Psychol.*, vol. 4, 2013, doi: 10.3389/fpsyg.2013.00080.

[22] A. E. Castaneda *et al.*, 'The Association between Discrimination and Psychological and Social Well-being: A Population-based Study of Russian, Somali and Kurdish Migrants in Finland', *Psychology and Developing Societies*, vol. 27, no. 2, pp. 270–292, Sep. 2015, doi: 10.1177/0971333615594054.

[23] M. Çetin, 'Effects of Religious Participation on Social Inclusion and Existential Well-Being Levels of Muslim Refugees and Immigrants in Turkey', *The International Journal for the Psychology of Religion*, vol. 29, no. 2, pp. 64–76, Apr. 2019, doi: 10.1080/10508619.2019.1580092.

[24] H.-C. Chang, 'Marital Power Dynamics and Well-Being of Marriage Migrants', *Journal of Family Issues*, vol. 37, no. 14, pp. 1994–2020, Nov. 2016, doi: 10.1177/0192513X15570317.

[25] S.-L. Cheung, W. P. Krijnen, C. P. Van Der Schans, and J. S. M. Hobbelen, 'Frailty, Quality of Life, and Loneliness of Aging in Native and Diasporic Chinese Adults', *The Journal of Frailty & Aging*, vol. 12, no. 3, pp. 221–230, Jul. 2023, doi: 10.14283/jfa.2022.27.

[26] M. Cleveland, R. Iyer, and B. J. Babin, 'Social media usage, materialism and psychological well-being among immigrant consumers', *Journal of Business Research*, vol. 155, p. 113419, Jan. 2023, doi: 10.1016/j.jbusres.2022.113419.

[27] C. L. Cobb *et al.*, 'Perceived discrimination and well-being among unauthorized Hispanic immigrants: The moderating role of ethnic/racial group identity centrality.', *Cultural Diversity & Ethnic Minority Psychology*, vol. 25, no. 2, pp. 280–287, Apr. 2019, doi: 10.1037/cdp0000227.

[28] G. J. Coffey, I. Kaplan, R. C. Sampson, and M. M. Tucci, 'The meaning and mental health consequences of long-term immigration detention for people seeking asylum', *Social Science & Medicine*, vol. 70, no. 12, pp. 2070–2079, Jun. 2010, doi: 10.1016/j.socscimed.2010.02.042.

[29] K. Cohen-Louck and M. Shechory-Bitton, 'Distress and wellbeing among 1.5-generation immigrants 3 decades after immigration to Israel', *Stress and Health*, vol. 38, no. 2, pp. 330–339, Apr. 2022, doi: 10.1002/smi.3094.

[30] J. M. Cramm and A. P. Nieboer, 'Aging perceptions matter for the well-being of elderly Turkish migrants, especially among the chronically ill', *BMC Geriatr*, vol. 18, no. 1, p. 229, Dec. 2018, doi: 10.1186/s12877-018-0902-4.

[31] J. M. Cramm and A. P. Nieboer, 'The importance of health behaviours and especially broader self-management abilities for older Turkish immigrants', *European Journal of Public Health*, vol. 28, no. 6, pp. 1087–1092, Dec. 2018, doi: 10.1093/eurpub/cky174.

[32] P. Cross, R. T. Edwards, B. Hounsome, and G. Edwards-Jones, 'Comparative assessment of migrant farm worker health in conventional and organic horticultural systems in the United Kingdom', *Science of The Total Environment*, vol. 391, no. 1, pp. 55–65, Feb. 2008, doi: 10.1016/j.scitotenv.2007.10.048.

[33] I. Cuellar, E. Bastida, and S. M. Braccio, 'Residency in the United States, Subjective Well-Being, and Depression in an Older Mexican-Origin Sample', *J Aging Health*, vol. 16, no. 4, pp. 447–466, Aug. 2004, doi: 10.1177/0898264304265764.

[34] N. Damri and H. Litwin, 'Minority population group status and QOL change: the case of older Israelis', *Eur J Ageing*, vol. 13, no. 4, pp. 299–309, Dec. 2016, doi: 10.1007/s10433-016-0396-x.

[35] F. D'Isanto, P. Fouskas, and M. Verde, 'Determinants of Well-being Among Legal and Illegal Immigrants: Evidence from South Italy', *Soc Indic Res*, vol. 126, no. 3, pp. 1109–1141, Apr. 2016, doi: 10.1007/s11205-015-0924-7.

[36] M. L. Dolezal *et al.*, 'Differences in Posttraumatic and Psychosocial Outcomes Among Refugees, Asylum Seekers, and Internally Displaced Persons', *J Nerv Ment Dis*, vol. 209, no. 1, pp. 28–34, Jan. 2021, doi: 10.1097/NMD.0000000000001248.

[37] N. Dou, L. E. Murray-Kolb, D. C. Mitchell, H. Melgar-Quiñonez, and M. Na, 'Food Insecurity and Mental Well-Being in Immigrants: A Global Analysis', *American Journal of Preventive Medicine*, vol. 63, no. 2, pp. 301–311, Aug. 2022, doi: 10.1016/j.amepre.2022.02.006.

[38] L. Dryjanska and C. Zlotnick, 'Acculturation of migrant latinos in a positive psychology framework.', *Hispanic Journal of Behavioral Sciences*, vol. 43, no. 3, pp. 155–173, 2021, doi: 10.1177/07399863211033745.

[39] I. Fassbender and B. Leyendecker, 'Socio-Economic Status and Psychological Well-Being in a Sample of Turkish Immigrant Mothers in Germany', *Front. Psychol.*, vol. 9, p. 1586, Oct. 2018, doi: 10.3389/fpsyg.2018.01586.

[40] A. García-Cid, L. Gómez-Jacinto, I. Hombrados-Mendieta, M. Millán-Franco, and G. Moscato, 'Discrimination and Psychosocial Well-Being of Migrants in Spain: The Moderating Role of Sense of Community', *Front. Psychol.*, vol. 11, p. 2235, Sep. 2020, doi: 10.3389/fpsyg.2020.02235.

[41] J. R. Goodkind, 'Effectiveness of a Community-Based Advocacy and Learning Program for Hmong Refugees', *American Journal of Community Psychology*, vol. 36, no. 3–4, pp. 387–408, Dec. 2005, doi: 10.1007/s10464-005-8633-z.

[42] K. Haagsman, V. Mazzucato, and B. B. Dito, 'Transnational families and the subjective well-being of migrant parents: Angolan and Nigerian parents in the Netherlands', *Ethnic and Racial Studies*, vol. 38, no. 15, pp. 2652–2671, Dec. 2015, doi: 10.1080/01419870.2015.1037783.

[43] J. Haj-Younes *et al.*, 'Changes in self-rated health and quality of life among Syrian refugees migrating to Norway: a prospective longitudinal study', *Int J Equity Health*, vol. 19, no. 1, p. 188, Dec. 2020, doi: 10.1186/s12939-020-01300-6.

[44] N. Hashemi, M. Marzban, B. Sebar, and N. Harris, ‘Perceived discrimination and subjective well-being among Middle Eastern migrants in Australia: The moderating role of perceived social support’, *Int J Soc Psychiatry*, vol. 67, no. 2, pp. 110–119, Mar. 2021, doi: 10.1177/0020764020940740.

[45] M. Hendriks and M. J. Burger, ‘Unsuccessful Subjective Well-Being Assimilation Among Immigrants: The Role of Faltering Perceptions of the Host Society’, *J Happiness Stud*, vol. 21, no. 6, pp. 1985–2006, Aug. 2020, doi: 10.1007/s10902-019-00164-0.

[46] J. Herrero, A. Fuente, and E. Gracia, ‘Covariates of Subjective well-being among Latin American immigrants in Spain: the role of social integration in the community’, *J. Community Psychol.*, vol. 39, no. 7, pp. 761–775, Sep. 2011, doi: 10.1002/jcop.20468.

[47] C. Hsieh, ‘Importance of Health and Relative Importance of Satisfaction with One’s Own Health: A Case of Frail Immigrant Older Adults’, *Soc Indic Res*, vol. 143, no. 1, pp. 81–93, May 2019, doi: 10.1007/s11205-018-1973-5.

[48] M. N. N. Htay, S. S. Latt, K. S. Maung, W. W. Myint, and S. Moe, ‘Mental Well-Being and Its Associated Factors Among Myanmar Migrant Workers in Penang, Malaysia’, *Asia Pac J Public Health*, vol. 32, no. 6–7, pp. 320–327, Sep. 2020, doi: 10.1177/1010539520940199.

[49] J. S. Jackson, I. Forsythe-Brown, and I. O. Govia, ‘Age Cohort, Ancestry, and Immigrant Generation Influences in Family Relations and Psychological Well-Being among Black Caribbean Family Members’, *Journal of Social Issues*, vol. 63, no. 4, pp. 729–743, Dec. 2007, doi: 10.1111/j.1540-4560.2007.00533.x.

[50] R. Katz, ‘Intergenerational Family Relations and Life Satisfaction Among Three Elderly Population Groups in Transition in the Israeli Multi-cultural Society’, *J Cross Cult Gerontol*, vol. 24, no. 1, pp. 77–91, Mar. 2009, doi: 10.1007/s10823-009-9092-z.

[51] A. Kearns, E. Whitley, M. Egan, C. Tabbner, and C. Tannahill, ‘Healthy Migrants in an Unhealthy City? The Effects of Time on the Health of Migrants Living in Deprived Areas of Glasgow’, *Int. Migration & Integration*, vol. 18, no. 3, pp. 675–698, Aug. 2017, doi: 10.1007/s12134-016-0497-6.

[52] J. Khatiwada, B. A. Muzembo, K. Wada, and S. Ikeda, ‘The effect of perceived social support on psychological distress and life satisfaction among Nepalese migrants in Japan’, *PLoS ONE*, vol. 16, no. 2, p. e0246271, Feb. 2021, doi: 10.1371/journal.pone.0246271.

[53] S. S. Klokgieters, T. G. Van Tilburg, D. J. H. Deeg, and M. Huisman, ‘Do religious activities among young–old immigrants act as a buffer against the effect of a lack of resources on well-being?’, *Aging & Mental Health*, vol. 23, no. 5, pp. 625–632, May 2019, doi: 10.1080/13607863.2018.1430739.

[54] G. Knies, A. Nandi, and L. Platt, ‘Life satisfaction, ethnicity and neighbourhoods: Is there an effect of neighbourhood ethnic composition on life satisfaction?’, *Social Science Research*, vol. 60, pp. 110–124, Nov. 2016, doi: 10.1016/j.ssresearch.2016.01.010.

[55] A. Kuehne, S. Huschke, and M. Bullinger, ‘Subjective health of undocumented migrants in Germany – a mixed methods approach’, *BMC Public Health*, vol. 15, no. 1, p. 926, Dec. 2015, doi: 10.1186/s12889-015-2268-2.

[56] N. Kushnirovich and R. Youngmann, ‘Paths to success of Israeli immigrants from different countries of origin’, *International Journal of Intercultural Relations*, vol. 60, pp. 51–59, Sep. 2017, doi: 10.1016/j.ijintrel.2017.07.002.

[57] L. C. H. Lai, R. A. Cummins, and A. L. D. Lau, ‘Cross-Cultural Difference in Subjective Wellbeing: Cultural Response Bias as an Explanation’, *Soc Indic Res*, vol. 114, no. 2, pp. 607–619, Nov. 2013, doi: 10.1007/s11205-012-0164-z.

[58] D. W. L. Lai, V. W. P. Lee, J. Li, and X. Dong, 'The Impact of Intergenerational Relationship on Health and Well-Being of Older Chinese Americans', *J American Geriatrics Society*, vol. 67, no. S3, Aug. 2019, doi: 10.1111/jgs.15893.

[59] T. V. Lats, P. Youngbin Kim, and D. A. Diekema, 'Acculturative Family Distancing, Religious Support, and Psychological Well-Being Among Young Adult Eastern European Immigrants in Western Washington', *PsiChiJournal*, vol. 21, no. 4, pp. 252–260, 2016, doi: 10.24839/2164-8204.JN21.4.252.

[60] E. Lee, S. I. Kim, K. Jung-Choi, and K. A. Kong, 'Household decision-making and the mental well-being of marriage-based immigrant women in South Korea', *PLoS ONE*, vol. 17, no. 2, p. e0263642, Feb. 2022, doi: 10.1371/journal.pone.0263642.

[61] G. Lee-Fong, L. B. Daniels, and L. M. Slifka, 'Spiritual intelligence and psychological well-being in refugees', *Mental Health, Religion & Culture*, vol. 25, no. 4, pp. 401–413, Apr. 2022, doi: 10.1080/13674676.2022.2032626.

[62] R. León-Pinilla, A. Soto-Rubio, and V. Prado-Gascó, 'Support and Emotional Well-Being of Asylum Seekers and Refugees in Spain', *IJERPH*, vol. 17, no. 22, p. 8365, Nov. 2020, doi: 10.3390/ijerph17228365.

[63] D. Levy and H. Itzhaky, 'Adjustment of women immigrants from Ethiopia: The contributing factors', *Social Development Issues, ,* vol. 38(2), no. 1–16, 2016, [Online]. Available: https://www.proquest.com/scholarly-journals/adjustment-women-immigrants-ethiopia-contributing/docview/1797694426/se-2?accountid=12372

[64] X. Lin, C. Bryant, J. Boldero, and B. Dow, 'Psychological well-being of older Chinese immigrants living in Australia: a comparison with older Caucasians', *International Psychogeriatrics*, vol. 28, no. 10, pp. 1671–1679, Oct. 2016, doi: 10.1017/S1041610216001010.

[65] J. Liu, W. Mao, M. Guo, L. Xu, I. Chi, and X. Dong, 'Loss of friends and psychological well-being of older Chinese immigrants', *Aging & Mental Health*, vol. 25, no. 2, pp. 323–331, Feb. 2021, doi: 10.1080/13607863.2019.1693967.

[66] M. Löfvander, A. Rosenblad, T. Wiklund, H. Bennström, and J. Leppert, 'A case–control study of self-reported health, quality-of-life and general functioning among recent immigrants and age- and sex-matched Swedish-born controls', *Scand J Public Health*, vol. 42, no. 8, pp. 734–742, Dec. 2014, doi: 10.1177/1403494814550175.

[67] J.-E. Lonnqvist, S. Leikas, T. A. Mahonen, and I. Jasinskaja-Lahti, 'The mixed blessings of migration: Life satisfaction and self-esteem over the course of migration.', *European Journal of Social Psychology*, vol. 45, no. 4, pp. 496–514, 2015, doi: 10.1002/ejsp.2105.

[68] D. Madi, M. Bobowik, M. Verkuyten, and N. Basabe, 'Social intergroup and temporal intrapersonal comparisons: Responses to perceived discrimination and protective mechanisms of eudaimonic well-being', *International Journal of Intercultural Relations*, vol. 86, pp. 74–84, Jan. 2022, doi: 10.1016/j.ijintrel.2021.10.007.

[69] K. Maier, K. Konaszewski, S. B. Skalski, A. Büssing, and J. Surzykiewicz, 'Spiritual Needs, Religious Coping and Mental Wellbeing: A Cross-Sectional Study among Migrants and Refugees in Germany', *IJERPH*, vol. 19, no. 6, p. 3415, Mar. 2022, doi: 10.3390/ijerph19063415.

[70] T. Mäki-Opas, R. Pieper, and M. Vaarama, 'Exploring the capability approach to quality of life in disadvantaged population groups', *Sci Rep*, vol. 12, no. 1, p. 15248, Sep. 2022, doi: 10.1038/s41598-022-18877-3.

[71] M. Vukčević Marković, A. Bobić, and M. Živanović, 'The effects of traumatic experiences during transit and pushback on the mental health of refugees, asylum seekers, and migrants', *European Journal of Psychotraumatology*, vol. 14, no. 1, p. 2163064, Dec. 2023, doi: 10.1080/20008066.2022.2163064.

[72] S. Martinez-Damia, V. Paloma, J. F. Luesia, E. Marta, and D. Marzana, ‘Community participation and subjective wellbeing among the immigrant population in Northern Italy: An analysis of mediators’, *American J of Comm Psychol*, vol. 71, no. 3–4, pp. 382–394, Jun. 2023, doi: 10.1002/ajcp.12652.

[73] K. Martynowska, T. Korulczyk, and P. J. Mamcarz, ‘Perceived stress and well-being of Polish migrants in the UK after Brexit vote’, *PLoS ONE*, vol. 15, no. 7, p. e0236168, Jul. 2020, doi: 10.1371/journal.pone.0236168.

[74] A. Matanov *et al.*, ‘Subjective quality of life in war-affected populations’, *BMC Public Health*, vol. 13, no. 1, p. 624, Dec. 2013, doi: 10.1186/1471-2458-13-624.

[75] E.-M. Merz and N. S. Consedine, ‘Ethnic group moderates the association between attachment and well-being in later life.’, *Cultural Diversity & Ethnic Minority Psychology*, vol. 18, no. 4, pp. 404–415, Oct. 2012, doi: 10.1037/a0029595.

[76] K. E. Miller *et al.*, ‘Supporting Syrian families displaced by armed conflict: A pilot randomized controlled trial of the Caregiver Support Intervention’, *Child Abuse & Neglect*, vol. 106, p. 104512, Aug. 2020, doi: 10.1016/j.chiabu.2020.104512.

[77] L. Monteiro and M. Haan, ‘The Life Satisfaction of Immigrants in Canada: Does Time Since Arrival Matter more than Income?’, *Int. Migration & Integration*, vol. 23, no. 3, pp. 1397–1420, Sep. 2022, doi: 10.1007/s12134-021-00899-x.

[78] A. Urzúa Morales, E. Delgado-Valencia, M. Rojas-Ballesteros, and A. Caqueo-Urízar, ‘Social Well-Being Among Colombian and Peruvian Immigrants in Northern Chile’, *J Immigrant Minority Health*, vol. 19, no. 5, pp. 1140–1147, Oct. 2017, doi: 10.1007/s10903-016-0416-0.

[79] A.-L. Morville, L.-K. Erlandsson, M. Eklund, B. Danneskiold-Samsøe, R. Christensen, and K. Amris, ‘Activity of Daily Living Performance amongst Danish Asylum Seekers: A cross-sectional study’, *torture*, vol. 24, no. 1, p. 16, Oct. 2018, doi: 10.7146/torture.v24i1.109720.

[80] A.-L. Morville, L.-K. Erlandsson, B. Danneskiold-Samsøe, K. Amris, and M. Eklund, ‘Satisfaction with daily occupations amongst asylum seekers in Denmark’, *Scandinavian Journal of Occupational Therapy*, vol. 22, no. 3, pp. 207–215, May 2015, doi: 10.3109/11038128.2014.982702.

[81] E. J. Murphy and R. Mahalingam, ‘Perceived congruence between expectations and outcomes: Implications for mental health among caribbean immigrants.’, *American Journal of Orthopsychiatry*, vol. 76, no. 1, pp. 120–127, 2006, doi: 10.1037/0002-9432.76.1.120.

[82] N. O. Nielsen, E. Benedikz, M. Dahl, J. Præstegaard, and M. Lindahl, ‘Health and wellbeing in refugee families from Syria resettled in Denmark’, *Journal of Migration and Health*, vol. 8, p. 100200, 2023, doi: 10.1016/j.jmh.2023.100200.

[83] C. Nikendei *et al.*, ‘Asylum seekers’ mental health and treatment utilization in a three months follow-up study after transfer from a state registration-and reception-center in Germany’, *Health Policy*, vol. 123, no. 9, pp. 864–872, Sep. 2019, doi: 10.1016/j.healthpol.2019.07.008.

[84] W. Pabilonia, S. P. Combs, and P. F. Cook, ‘Knowledge and quality of life in female torture survivors’, *Torture*, vol. 20, no. 1, pp. 4–22, 2010.

[85] V. Paloma, M. García-Ramírez, and C. Camacho, ‘Well-Being and Social Justice Among Moroccan Migrants in Southern Spain’, *American J of Comm Psychol*, vol. 54, no. 1–2, pp. 1–11, Sep. 2014, doi: 10.1007/s10464-014-9663-1.

[86] A. Paparusso, ‘Studying Immigrant Integration Through Self-Reported Life Satisfaction in the Country of Residence’, *Applied Research Quality Life*, vol. 14, no. 2, pp. 479–505, Apr. 2019, doi: 10.1007/s11482-018-9624-1.

[87] C. Perera *et al.*, 'A brief psychological intervention for improving the mental health of Venezuelan migrants and refugees: A mixed-methods study', *SSM - Mental Health*, vol. 2, p. 100109, Dec. 2022, doi: 10.1016/j.ssmmh.2022.100109.

[88] D. R. Du Plooy, A. Lyons, and E. S. Kashima, 'The Effect of Social Support on Psychological Flourishing and Distress Among Migrants in Australia', *J Immigrant Minority Health*, vol. 21, no. 2, pp. 278–289, Apr. 2019, doi: 10.1007/s10903-018-0745-2.

[89] C. Prapas and V. Mavreas, 'The Relationship Between Quality of Life, Psychological Wellbeing, Satisfaction with Life and Acculturation of Immigrants in Greece', *Cult Med Psychiatry*, vol. 43, no. 1, pp. 77–92, Mar. 2019, doi: 10.1007/s11013-018-9598-3.

[90] M. Raffaelli, S. P. Tran, A. R. Wiley, M. Galarza-Heras, and V. Lazarevic, 'Risk and Resilience in Rural Communities: The Experiences of Immigrant Latina Mothers', *Family Relations*, vol. 61, no. 4, pp. 559–570, Oct. 2012, doi: 10.1111/j.1741-3729.2012.00717.x.

[91] M. R. Read-Wahidi and J. A. DeCaro, 'Guadalupan Devotion as a Moderator of Psychosocial Stress among Mexican Immigrants in the Rural Southern United States', *Med Anthropol Q*, vol. 31, no. 4, pp. 572–591, Dec. 2017, doi: 10.1111/maq.12372.

[92] S. Regev and V. Slonim-Nevo, 'Sorrow shared is halved? War trauma experienced by others and mental health among Darfuri asylum seekers', *Psychiatry Research*, vol. 273, pp. 475–480, Mar. 2019, doi: 10.1016/j.psychres.2019.01.049.

[93] L. Remennick, 'Immigration, Gender, and Psychosocial Adjustment: A Study of 150 Immigrant Couples in Israel', *Sex Roles*, vol. 53, no. 11–12, pp. 847–863, Dec. 2005, doi: 10.1007/s11199-005-8297-z.

[94] A. L. Roy and E. B. Godfrey, 'RELATIONSHIPS BETWEEN FAMILY AND NEIGHBORHOOD INCOME AND FIRST-GENERATION LATINO ADULTS' DEPRESSIVE SYMPTOMS AND Well-BEING', *Journal Community Psychology*, vol. 44, no. 7, pp. 856–871, Sep. 2016, doi: 10.1002/jcop.21813.

[95] T. A. Ryabichenko and N. Lebedeva, 'Assimilation or integration: Similarities and differences between acculturation attitudes of migrants from Central Asia and Russians in Central Russia', *Psych. Rus.*, vol. 9, no. 1, pp. 98–111, 2016, doi: 10.11621/pir.2016.0107.

[96] R. Sander, H. Laugesen, S. Skammeritz, E. L. Mortensen, and J. Carlsson, 'Interpreter-mediated psychotherapy with trauma-affected refugees – A retrospective cohort study', *Psychiatry Research*, vol. 271, pp. 684–692, Jan. 2019, doi: 10.1016/j.psychres.2018.12.058.

[97] L. Schiltz and J. Schiltz, 'When the Foundations of Life have been Upset…. An Integrated Clinical and Experimental Study with Refugees and Asylum Seekers', *Arch Psych Psych*, vol. 15, no. 2, pp. 53–62, Jun. 2013, doi: 10.12740/APP/17496.

[98] K. M. Scottham and R. H. Dias, 'Acculturative Strategies and the Psychological Adaptation of Brazilian Migrants to Japan', *Identity*, vol. 10, no. 4, pp. 284–303, Oct. 2010, doi: 10.1080/15283488.2010.523587.

[99] H. Simkin, 'The Centrality of Events, Religion, Spirituality, and Subjective Well-Being in Latin American Jewish Immigrants in Israel', *Front. Psychol.*, vol. 11, p. 576402, Sep. 2020, doi: 10.3389/fpsyg.2020.576402.

[100] S. Stupar, F. J. R. Van De Vijver, and J. R. J. Fontaine, 'Emotional suppression and well-being in immigrants and majority group members in the Netherlands', *Int J Psychol*, vol. 49, no. 6, pp. 503–507, Dec. 2014, doi: 10.1002/ijop.12040.

[101] C. M. R. Sulaiman-Hill and S. C. Thompson, 'Afghan and Kurdish refugees, 8–20 years after resettlement, still experience psychological distress and challenges to well

being', *Australian and New Zealand Journal of Public Health*, vol. 36, no. 2, pp. 126–134, Apr. 2012, doi: 10.1111/j.1753-6405.2011.00778.x.

[102] M. Taloyan, J. Sundquist, and A. Al-Windi, 'The impact of ethnicity and self-reported health on psychological well-being: a comparative study of Kurdish-born and Swedish-born people.', *Nordic journal of psychiatry*, vol. 62, no. 5, pp. 392–8, 2008, doi: 10.1080/08039480801984263.

[103] E. Tartakovsky and S. D. Walsh, 'Factors affecting the psychological well-being of immigrants: The role of group self-appraisal, social contacts, and perceived ethnic density.', *Cultural Diversity & Ethnic Minority Psychology*, vol. 26, no. 4, pp. 592–603, Oct. 2020, doi: 10.1037/cdp0000333.

[104] P. Tinghög, S. Al-Saffar, J. Carstensen, and L. Nordenfelt, 'The Association of Immigrant- and Non-Immigrant-Specific Factors With Mental Ill Health Among Immigrants in Sweden', *Int J Soc Psychiatry*, vol. 56, no. 1, pp. 74–93, Jan. 2010, doi: 10.1177/0020764008096163.

[105] L. K. Tip, R. Brown, L. Morrice, M. Collyer, and M. J. Easterbrook, 'Improving Refugee Well-Being With Better Language Skills and More Intergroup Contact', *Social Psychological and Personality Science*, vol. 10, no. 2, pp. 144–151, Mar. 2019, doi: 10.1177/1948550617752062.

[106] K. Topal, E. Eser, I. Sanberk, E. Bayliss, and E. Saatci, 'Challenges in access to health services and its impact on quality of life: a randomised population-based survey within Turkish speaking immigrants in London', *Health Qual Life Outcomes*, vol. 10, no. 1, p. 11, 2012, doi: 10.1186/1477-7525-10-11.

[107] S. Toselli, N. Rinaldo, M. G. Caccialupi, and E. Gualdi-Russo, 'Psychosocial Indicators in North African Immigrant Women in Italy', *J Immigrant Minority Health*, vol. 20, no. 2, pp. 431–440, Apr. 2018, doi: 10.1007/s10903-017-0562-z.

[108] A. Urzúa, R. Ferrer, V. Canales Gaete, D. Núñez Aragón, I. Ravanal Labraña, and B. Tabilo Poblete, 'The influence of acculturation strategies in quality of life by immigrants in Northern Chile', *Qual Life Res*, vol. 26, no. 3, pp. 717–726, Mar. 2017, doi: 10.1007/s11136-016-1470-8.

[109] A. Urzúa *et al.*, 'The mediating effect of self-esteem on the relationship between perceived discrimination and psychological well-being in immigrants', *PLoS ONE*, vol. 13, no. 6, p. e0198413, Jun. 2018, doi: 10.1371/journal.pone.0198413.

[110] A. Urzúa, D. Henríquez, A. Caqueo-Urízar, and R. Landabur, 'Ethnic Identity and Collective Self-Esteem Mediate the Effect of Anxiety and Depression on Quality of Life in a Migrant Population', *IJERPH*, vol. 19, no. 1, p. 174, Dec. 2021, doi: 10.3390/ijerph19010174.

[111] A. Urzúa *et al.*, 'Ethnic Identity as a Mediator of the Relationship between Discrimination and Psychological Well-Being in South—South Migrant Populations', *IJERPH*, vol. 18, no. 5, p. 2359, Feb. 2021, doi: 10.3390/ijerph18052359.

[112] A. K. Uskul and E. Greenglass, 'Psychological wellbeing in a Turkish-Canadian sample', *Anxiety, Stress & Coping*, vol. 18, no. 3, pp. 269–278, Sep. 2005, doi: 10.1080/10615800500205983.

[113] E. Uygun, 'The Life Condition of Syrian Asylum Seekers in Turkey and the Effect of These Conditions on the Desire to Migrate to Europe', *Psychiatry Investig*, vol. 17, no. 1, pp. 55–60, Jan. 2020, doi: 10.30773/pi.2018.0275.

[114] E. Uygun, 'The Relationship Between the Types of Traumatic Events and Well-Being, Post-Traumatic Stress Levels and Gender Differences in Syrian Patients: A Cross-Sectional Controlled Study', *J Immigrant Minority Health*, vol. 23, no. 6, pp. 1232–1240, Dec. 2021, doi: 10.1007/s10903-020-01097-0.

[115] S. Var, S. Poyrazli, and K. M. Grahame, 'Personal Well-being and Overall Satisfaction of Life of Asian Immigrant and Refugee Women', *J. Asia Pac. Couns.*, vol. 3, no. 1, pp. 77–90, Jun. 2013, doi: 10.18401/2013.3.1.6.

[116] P. J. Ramos Villagrasa and A. L. García Izquierdo, 'The price of working abroad: Well-being among immigrant and native workers', *Ansiedad y Estrés*, vol. 24, no. 2–3, pp. 125–130, Jul. 2018, doi: 10.1016/j.anyes.2018.08.001.

[117] N. Vohra and J. Adair, 'Life Satisfaction of Indian Immigrants in Canada', *Psychology and Developing Societies*, vol. 12, no. 2, pp. 109–138, Sep. 2000, doi: 10.1177/097133360001200201.

[118] L. Walther, L. M. Fuchs, J. Schupp, and C. Von Scheve, 'Living Conditions and the Mental Health and Well-being of Refugees: Evidence from a Large-Scale German Survey', *J Immigrant Minority Health*, vol. 22, no. 5, pp. 903–913, Oct. 2020, doi: 10.1007/s10903-019-00968-5.

[119] J. Wångdahl, P. Lytsy, L. Mårtensson, and R. Westerling, 'Poor health and refraining from seeking healthcare are associated with comprehensive health literacy among refugees: a Swedish cross-sectional study', *Int J Public Health*, vol. 63, no. 3, pp. 409–419, Apr. 2018, doi: 10.1007/s00038-017-1074-2.

[120] Z. Xiao, J. Lee, and W. Liu, 'Korean and Vietnamese immigrants are not the same: Health literacy, health status, and quality of life', *Journal of Human Behavior in the Social Environment*, vol. 30, no. 6, pp. 711–729, Aug. 2020, doi: 10.1080/10911359.2020.1740852.

[121] L. Xu, N. L. Fields, B. C. Tonui, and T. Vasquez-White, 'Empowering older Chinese immigrant volunteers: A pilot study of a psychoeducational intervention for foster grandparents', *SSM - Mental Health*, vol. 2, p. 100111, Dec. 2022, doi: 10.1016/j.ssmmh.2022.100111.

[122] L. Yakhnich and H. Ben-Zur, 'Personal resources, appraisal, and coping in the adaptation process of immigrants from the Former Soviet Union.', *American Journal of Orthopsychiatry*, vol. 78, no. 2, pp. 152–162, Apr. 2008, doi: 10.1037/0002-9432.78.2.152.

[123] A. C. Yalim, 'The Impacts of Contextual Factors on Psychosocial Wellbeing of Syrian Refugees: Findings from Turkey and the United States', *Journal of Social Service Research*, vol. 47, no. 1, pp. 104–117, Jan. 2021, doi: 10.1080/01488376.2020.1717717.

[124] R. Youngmann and N. Kushnirovich, 'Income as a resilience factor for the impact of discrimination and institutional unfairness on minorities' emotional well-being', *Social Science Research*, vol. 91, p. 102462, Sep. 2020, doi: 10.1016/j.ssresearch.2020.102462.

[125] W. Zhang, S. Liu, K. Zhang, and B. Wu, 'Neighborhood Social Cohesion, Resilience, and Psychological Well-Being Among Chinese Older Adults in Hawai'i', *The Gerontologist*, vol. 60, no. 2, pp. 229–238, Feb. 2020, doi: 10.1093/geront/gnz104.

[126] X. Zou, B. J. Hall, M. Xiong, and C. Wang, 'Post-migration well-being of Sub-Saharan Africans in China: a nationwide cross-sectional survey', *Qual Life Res*, vol. 30, no. 4, pp. 1025–1035, Apr. 2021, doi: 10.1007/s11136-020-02663-7.

[127] R. A. Cummins, R. Eckersley, J. Pallant, J. Van Vugt, and R. Misajon, 'Developing a National Index of Subjective Wellbeing: The Australian Unity Wellbeing Index', *Social Indicators Research*, vol. 64, no. 2, pp. 159–190, 2003, doi: 10.1023/A:1024704320683.

[128] International Wellbeing Group, *Personal Wellbeing Index: 5th Edition.* Melbourne: Australian Centre on Quality of Life, Deakin University, 2013. [Online]. Available: http://www.deakin.edu.au/research/acqol/instruments/wellbeing-index/index.php

[129] E. Diener, R. A. Emmons, R. J. Larsen, and S. Griffin, 'The Satisfaction With Life Scale', *Journal of Personality Assessment*, vol. 49, no. 1, pp. 71–75, Feb. 1985, doi: 10.1207/s15327752jpa4901_13.

[130] D. P. Goldberg and P. Williams, *A user's guide to the General Health Questionnaire*, Reprint. Great Britain: GL Assessment, 1991.

[131] C. D. Ryff, 'Happiness is everything, or is it? Explorations on the meaning of psychological well-being.', *Journal of Personality and Social Psychology*, vol. 57, no. 6, pp. 1069–1081, Dec. 1989, doi: 10.1037/0022-3514.57.6.1069.

[132] C. D. Ryff and C. L. M. Keyes, 'The structure of psychological well-being revisited.', *Journal of Personality and Social Psychology*, vol. 69, no. 4, pp. 719–727, 1995, doi: 10.1037/0022-3514.69.4.719.

[133] The WHOQOL Group, 'The Development of the World Health Organization Quality of Life Assessment Instrument (the WHOQOL)', in *Quality of Life Assessment: International Perspectives*, J. Orley and W. Kuyken, Eds., Berlin, Heidelberg: Springer Berlin Heidelberg, 1994, pp. 41–57. doi: 10.1007/978-3-642-79123-9_4.

[134] The WHOQOL Group, 'Development of the WHOQOL: Rationale and Current Status', *International Journal of Mental Health*, vol. 23, no. 3, pp. 24–56, Sep. 1994, doi: 10.1080/00207411.1994.11449286.

[135] The WHOQOL Group, 'The World Health Organization quality of life assessment (WHOQOL): Position paper from the World Health Organization', *Social Science & Medicine*, vol. 41, no. 10, pp. 1403–1409, Nov. 1995, doi: 10.1016/0277-9536(95)00112-K.

[136] WHO, *WHOQoL User Manual (WHO/MNH/MHP/98.4.Rev.1)*. Geneva, Switzerland: WHO, 1998.

[137] C. W. Topp, S. D. Østergaard, S. Søndergaard, and P. Bech, 'The WHO-5 Well-Being Index: A Systematic Review of the Literature', *Psychother Psychosom*, vol. 84, no. 3, pp. 167–176, 2015, doi: 10.1159/000376585.

[138] WHO-EURO, *Wellbeing measures in primary health care/ the DEPCARE project (EUR/ICP/QCPH 05 01 03/ E60246*. Stockholm, Sweden: WHO, 1998.

[139] L. B. Mokkink *et al.*, 'The COSMIN study reached international consensus on taxonomy, terminology, and definitions of measurement properties for health-related patient-reported outcomes', *Journal of Clinical Epidemiology*, vol. 63, no. 7, pp. 737–745, Jul. 2010, doi: 10.1016/j.jclinepi.2010.02.006.
